# Analysis of cytosine deamination events in excision repair sequencing reads reveals mechanisms of incision site selection in NER

**DOI:** 10.1093/nar/gkad1195

**Published:** 2023-12-18

**Authors:** Benjamin Morledge-Hampton, Ananth Kalyanaraman, John J Wyrick

**Affiliations:** School of Molecular Biosciences, Washington State University, Pullman, WA 99164, USA; School of Electrical Engineering and Computer Science, Washington State University, Pullman, WA 99164, USA; School of Molecular Biosciences, Washington State University, Pullman, WA 99164, USA

## Abstract

Nucleotide excision repair (NER) removes helix-distorting DNA lesions and is therefore critical for genome stability. During NER, DNA is unwound on either side of the lesion and excised, but the rules governing incision site selection, particularly in eukaryotic cells, are unclear. Excision repair-sequencing (XR-seq) sequences excised NER fragments, but analysis has been limited because the lesion location is unknown. Here, we exploit accelerated cytosine deamination rates in UV-induced CPD (cyclobutane pyrimidine dimer) lesions to precisely map their locations at C to T mismatches in XR-seq reads, revealing general and species-specific patterns of incision site selection during NER. Our data indicate that the 5′ incision site occurs preferentially in HYV (i.e. not G; C/T; not T) sequence motifs, a pattern that can be explained by sequence preferences of the XPF-ERCC1 endonuclease. In contrast, the 3′ incision site does not show strong sequence preferences, once truncated reads arising from mispriming events are excluded. Instead, the 3′ incision is partially determined by the 5′ incision site distance, indicating that the two incision events are coupled. Finally, our data reveal unique and coupled NER incision patterns at nucleosome boundaries. These findings reveal key principles governing NER incision site selection in eukaryotic cells.

## Graphical Abstract

**Graphical Abstract:**
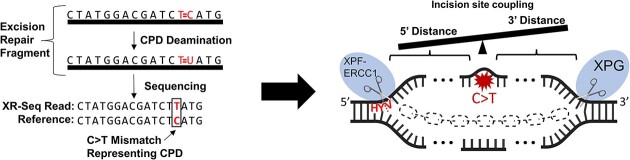

## Introduction

Nucleotide excision repair (NER) plays a critical role in maintaining genome stability by excising DNA fragments containing helix distorting lesions, such as ultraviolet (UV) photoproducts and cisplatin adducts (1,2). Individuals with inherited defects in NER genes, which cause the disease xeroderma pigmentosum (XP), have severe UV-sensitivity and greater than 1000-fold higher risk of developing skin cancer, highlighting the importance of NER to human health (3). NER is composed of two sub-pathways, global genome NER (GG-NER) and transcription-coupled NER (TC-NER), which repair a wide array of helix distorting lesions but differ in the repair proteins they use to detect damage. GG-NER relies on the XPC protein to directly identify DNA lesions that disrupt the normal B-form helical structure (4–7), whereas TC-NER uses CSA and CSB to detect RNA polymerase stalling at bulky, helix-distorting lesions (8–10). Following damage recognition, the two pathways converge as both recruit the TFIIH complex for helix unwinding and lesion verification. The XPB ATP-dependent DNA translocase and XPD helicase subunits of TFIIH unwind the DNA on the 5′ and 3′ sides of the lesion, respectively (11–13). XPD is also responsible for verifying that a lesion is present (6,14). Following DNA unwinding, the XPF-ERCC1 and XPG structure-specific endonucleases are recruited to cut at the 5′ and 3′ ends of the damaged strand, respectively (15). After the damaged strand is excised, DNA synthesis and ligation complete repair.

Although the biochemistry of the NER reaction has been studied for decades, a number of key aspects surrounding the incision reactions have yet to be elucidated. It is known that excised NER fragments in human cells range from about 25 to 30 nucleotides in length, with the lesion asymmetrically located near the 3′ end (16–18), but the molecular mechanisms that regulate this incision pattern in cells are largely unclear. Previous work has shown that the XPF-ERCC1 (19–22) and XPG (23) endonucleases cut near the junction between double-stranded and single-stranded DNA. Hence, incision site selection could be, in principle, regulated by the XPB and XPD subunits, which are required to unwind the DNA and create these junctions (11,13). Alternatively, incision site selection may be due to sequence specificities of the endonucleases themselves. Several studies have reported a sequence specificity for the purified XPF-ERCC1 endonuclease acting on a model DNA substrate, including a preference for pyrimidines immediately 5′ of the incision site (19,21,24). However, whether XPF-ERCC1 shows similar sequence preferences while it is complexed with TFIIH and other repair factors during NER is unclear. It is also unclear whether these *in vitro* findings can explain variations in NER fragment length and incision site choice *in vivo*. Finally, whether the location of the 5′ incision, which occurs first during NER (15,25), influences subsequent 3′ incision site choice is unknown.

Recently, the eXcision Repair sequencing (XR-seq) protocol was developed to sequence excised NER fragments directly from cells (16,26). XR-seq has been used to investigate cellular NER activity for a variety of DNA lesions, including UV-induced cyclobutane pyrimidine dimers (CPDs), 6–4 pyrimidine-pyrimidone photoproducts (6–4PPs), and cisplatin adducts from a myriad of species (e.g. human, *Arabidopsis*, yeast) (16,27–31). This protocol has been leveraged to better understand how NER operates throughout the genome as a whole and with respect to features like nucleosomes (32–34) and transcription factor binding sites (33,35–39). Furthermore, it has provided a wealth of data on the fragments themselves, such as information on size and nucleotide frequencies (27). However, detailed analysis of incision site selection during NER was previously not feasible due the inability to precisely locate the lesion in the repair fragments. While lesion position can sometimes be inferred from sequence biases at specific locations in XR-seq reads, this results only in an estimate of positioning across the population of fragments and cannot be used to precisely specify lesion position in individual fragments. A more precise method of lesion mapping within individual XR-seq reads would allow for detailed characterization of NER incision patterns in cells.

In this work, we describe methods for precisely mapping CPD lesions at single-nucleotide resolution within XR-seq fragments by exploiting characteristic C > T and CC > TT mismatches arising from accelerated cytosine deamination rates in CPDs. In undamaged, double-stranded DNA, cytosines deaminate with a half-life in the tens of thousands of years (40), but in CPDs, this half-life is shortened to as little as 24 h or less (41–43). Cytosine deaminates to uracil (or thymine if methylated), so aligning deaminated XR-seq reads to a reference genome results in a C to T (C > T) or CC > TT mismatch which can be used to pinpoint CPD positions at single-nucleotide resolution.

Here, we use this method to characterize species-specific differences in the variability of the 5′ and 3′ incision sites. Additionally, we identify a conserved sequence motif associated with the 5′ incision site that can be explained by sequence preferences of the XPF-ERCC1 endonuclease. The 3′ incision site does not show any consistent sequence bias, apart from a TGG enrichment most likely caused by adapter mispriming. Instead, more detailed analysis revealed that in the eukaryotic NER reaction, the two incision sites are coupled, such that the location of the 3′ incision site is partially determined by the location of the 5′ incision site. Finally, analysis of NER incision patterns in chromatin revealed significant variations in incision site distance specifically at nucleosome boundaries.

## Materials and methods

### Calling CPD positions from alignment mismatches

XR-seq reads from previous studies (see data availability section) were aligned using bowtie v2.4.5 with default parameters using the hg19 (human), TAIR10.1 (*Arabidopsis*), sacCer3 (yeast), and K-12 MG1655 (*Escherichia coli*) genome assemblies. In the resulting sam files, reads with mismatches to the reference genome were retrieved and the locations and sequences of the mismatches were recorded. Reads were filtered based on the following criteria: (i) reads with insertions or deletions were removed; (ii) reads with greater than one mismatch were removed (analysis of tandem CC > TT events were analyzed separately, see below); (iii) reads containing any ‘N’ nucleotides were removed; (iv) reads that did not fall within a species-specific read length range were removed.

After validation, all non-C > T mismatches were removed and C > T mismatch position frequencies were calculated relative to 3′ ends of reads, stratified by read length. Valid CPD positions for each read length were called from these frequencies by calculating a background distribution from the ten positions closest to the 5′ incision site (where no CPDs are expected) and finding positions with frequencies elevated at least four standard deviations above the background mean.

### Species-specific read length filtering

Valid read length ranges were determined by binning all aligned sequencing reads for a given species by read length and finding the nucleotide position relative to the 3′ read end with the highest dipyrimidine frequency in each bin. These frequencies were multiplied by the number of reads for their respective bins to generate a score for that read length. For each species, we established a continuous range of suitable read lengths by defining borders outside of which the score declined sharply, indicating less common read lengths, unreliable lesion positioning, or both. Where there was uncertainty in defining these borders, longer read lengths were favored due to the possibility of excision fragment degradation in shorter reads. In cases where multiple distinct ranges appeared viable (i.e. a bimodal distribution of scores was present) we chose the range based on the expected read length determined by previous experimental work. This was necessary for *Arabidopsis* (44) and yeast (45). The ranges used were 24–30 bp for *Arabidopsis*, 11–13 bp for *E. coli*, 22–30 bp for human, and 22–25 bp for yeast.

### PCR reactions to test for adapter mispriming

Three template sequences were selected with varying degrees of homology to the 3′ XR-seq adapter sequence, and primer sequences were adapted from the XR-seq protocol (26) (See Supplementary Table S1). The template sequences were based off of a short XR-seq read with a flanking TGG sequence. The three template sequences along with a no-template control were amplified using two separate polymerases, EconoTaq and Kappa HiFi. Both reactions contained 1.25 μM forward primer, 1.25 μM reverse primer, and 50 nM template. The EconoTaq reaction additionally contained 25 U/nL EconoTaq polymerase (30031-1, Biosearch Technologies), 1× EconoTaq buffer, and 200 μM dNTPs (N0447L, NEB). The Kapa HiFi reaction additionally contained 1× KAPA HiFi HotStart ReadyMix (07958897001, Roche Diagnostics). For both reactions, thermocycler conditions were as follows: (i) 95°C for 3 min, (ii) 98°C for 20 s, (iii) 62°C for 15 s, (iv) 72°C for 15 s. PCR reactions were run for a total of 31 cycles. PCR products were run on 2% agarose gels and visualized by staining with SYBR Safe.

### Filtering reads with 3′ TGG sequences

To remove reads which were potentially the product of PCR mispriming, all aligned read sequences were extended by three or more base pairs on the 3′ end using the surrounding genomic context. In order to extend these sequences, the genomic coordinates returned by bowtie2 after alignment were expanded by 3 nucleotides on either end and recorded in bed format. This bed file was then passed to the BEDTools (46) getfasta command alongside the ‘-s’ flag to return the expanded sequences while maintaining strand specificity. Using the resulting fasta file of expanded sequences, reads with TGG sequences flanking the 3′ read ends were identified and removed.

### Calculating relative 3′ and 5′ incision site contributions to read length variability

For each species analyzed, the mean distance between C > T mismatches and each incision site was calculated at every valid read length (see section on species-specific read length filtering). These distances were used to generate ‘position difference’ values by calculating the difference between the mean distance for the read length in question and the mean distance for the minimum read length. Position differences at each read length were then weighted based on the relative frequencies of each read length, stratified by the relevant incision site used to calculate distance (3′ or 5′) and summed across the two resulting groups. These sums were compared directly to one another to determine relative contributions to read length variability by each incision site.

### Sequence logo generation and GC content calculation

Sequence logos were generated using WebLogo v3.7.12 (47) with the CreateWebLogo.py script and SequenceLogoGeneration.ipynb notebook which can be found with the rest of the code at https://github.com/bmorledge-hampton19/deamination_determination. In short, input data consisted of aligned XR-seq reads with 10 base pairs of flanking genomic DNA on either side (derived using the BEDTools(46) getfasta command). Reads with 3′ flanking TGG sequences were filtered out. GC content was calculated on the input sequences for each sequence logo, and these values were used to normalize the sequence logo with the ‘–composition’ argument. This GC content was also used to calculate expected nucleotide frequencies (e.g. the frequency of TGG sequences). All other used parameters were merely cosmetic (i.e. they did not change the information content at any position in the logo) and can be found in CreateWebLogo.py.

### Identifying and mapping reads with tandem deamination events

In order to align reads with tandem deamination events, (i.e. CC > TT mismatches), input read sequences were searched for any and all TT dinucleotide sequences. Wherever a TT sequence was found, a putative revertant sequence was generated with the TT sequence replaced by CC. All putative revertant sequences for a given read were grouped together along with the original sequence and passed to Bowtie2 for alignment using exact matching. Exact matching was achieved in Bowtie v2.4.5 using the following parameters:

–reorder –no-1mm-upfront –no-unal –score-min C,0,0 -k 2

In cases where: (i) only one putative revertant sequence aligned, (ii) that sequence aligned exactly once and (iii) the original read sequence did not align, the original sequence was said to contain a CC > TT mismatch at the position indicated by the putative revertant sequence. CPDs were called from these tandem deamination events using the same read length, position, and 3′ TGG filtering constraints as single C > T mismatches.

### Quantifying incision site coupling

Cut site distances were calculated directly from mismatch positions and were defined as inclusive of the mismatched base in single mismatches or inclusive of the half base position between mismatched bases in tandem mismatches. The slope of the data was derived from a standard linear regression model, and the coupling percentage was defined as –100% times the slope for the linear regression of 5′ cut site distance versus 3′ cut site distance. Shuffling of 5′ cut site distances was achieved by taking a random sample of the available 5′ cut site distances without replacement. Read lengths were recalculated after shuffling. Reads with non-C > T mismatches were subjected to the same filtering constraints as those with C > T mismatches. For filtering based on enriched mismatch positions, non-C > T mismatch positions were filtered using C > T enrichment levels in the corresponding timepoint and cell type.

### Nucleosome-proximal analysis

∼12 million nucleosome dyad positions were called from a previously generated MNase map (48) using a high-resolution pipeline designed for nucleosome periodicity analysis (34) with the following modifications: Dyad positions were called throughout the genome instead of being constrained to mappable genic regions. Dyad positions were lifted over from the hg18 to the hg19 assembly using the UCSC genome browser LiftOver tool (49). Dyad positions were filtered using the Duke and DAC blacklisted regions (see data availability section) using a custom Perl script. Briefly, dyad positions were extended by 73 nucleotides on either side, and any that overlapped with the blacklisted regions were excluded. Reads with C > T mismatches positioned within 500 bp of a called nucleosome dyad were recorded and binned based on the distance between the mismatch and the dyad. Cut site distances were defined relative to each mismatch while maintaining binning by relative mismatch position. Data was strand aligned so that all 5′ incisions occurred in the negative direction and all 3′ incisions occurred in the positive direction, relative to the mismatch. Positions with significantly higher/lower mean cut site distances were identified using a p-value cutoff of 5e-5 for a two-tailed student's t-test comparing the distribution of cut site distances at a given bin to the mean cut site distance across all bins.

### 3′ NER endonuclease alignment

Sequences for the 3′ NER endonucleases were obtained from UniProt (50) using the following accession IDs. Human: P28715, *Arabidopsis*: Q9ATY5, Yeast: P07276. The alignment was produced by the Clustal Omega algorithm (51). Boxshade formatting was achieved using the following website: https://junli.netlify.app/apps/boxshade/. Key catalytic regions were determined using a previous alignment detailing catalytic residues and XP mutants (52). Interactions with XPD were determined using published data on a model of XPG interacting with TFIIH (53).

### Code availability

The code used for this analysis can be found through Zenodo at https://doi.org/10.5281/zenodo.10211636. Future updates to the analysis pipeline, as well as an explanation of how to implement this code base, can be found at https://github.com/bmorledge-hampton19/deamination_determination.

## Results

### C to T mismatches in aligned XR-seq reads indicate location of CPDs

**Figure 1.**
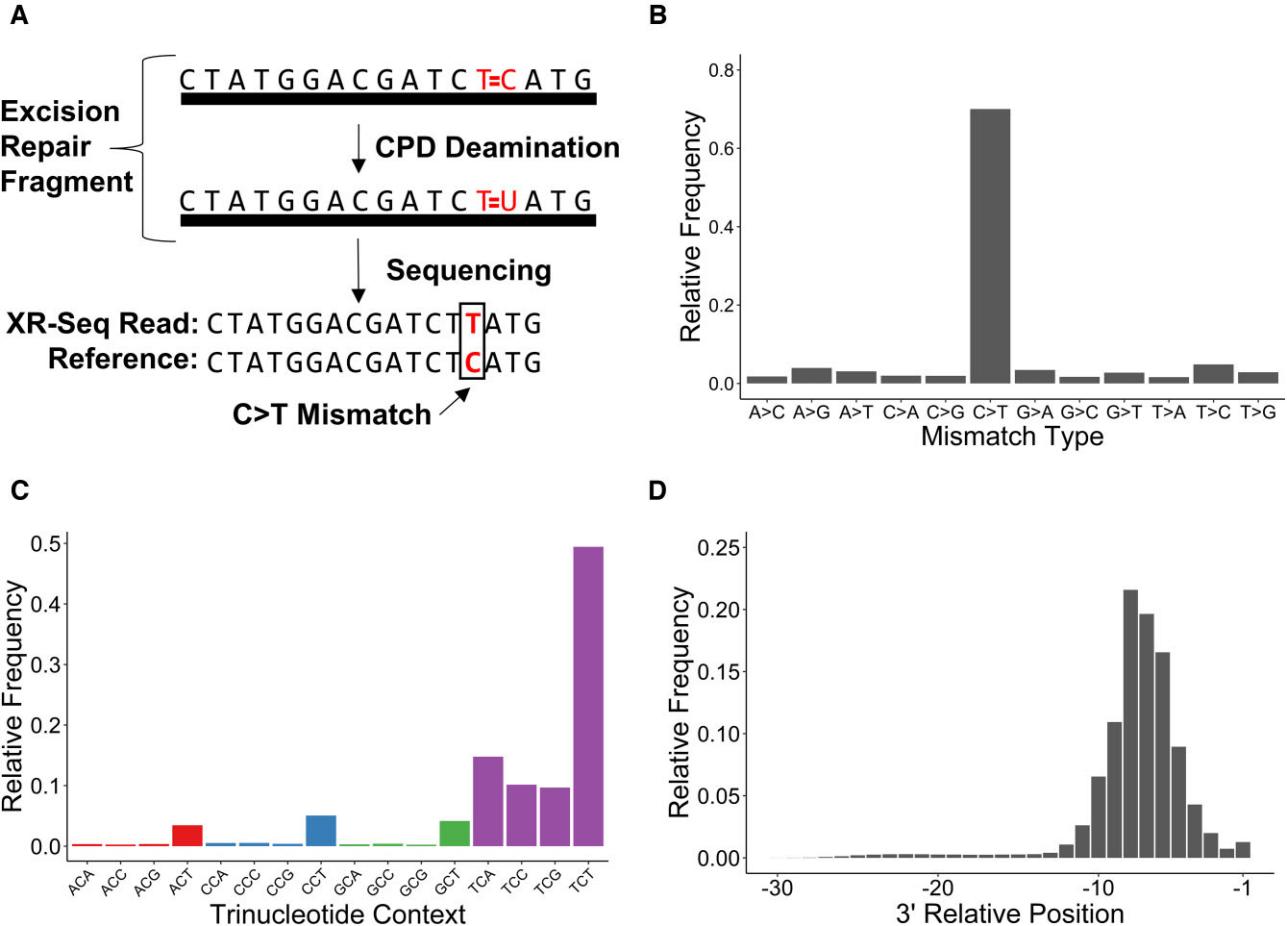
C > T mismatches in aligned XR-seq reads are indicative of CPD positions. (**A**) Schematic of the process by which deaminated cytosines are used to identify CPD positions. The bases in red with two lines between them represent the CPD. (**B**) Frequencies of each of the 12 single nucleotide mismatch types. (**C**) Frequencies of each of the 16 trinucleotide contexts for C > T mismatches. (**D**) Frequencies of the occurrence of C > T mismatches at read positions relative to the 3′ end. The –1 position on the x-axis indicates the first nucleotide before the 3′ cut site of the XR-seq read. Data for plots B–D were derived from aligned CPD XR-seq reads from NHF1 cells after 1 hour of repair.

To test whether we could detect deaminated CPDs in XR-seq reads, we leveraged data from previous studies (16,27,29–31,54) to identify and compile mismatches in XR-seq reads aligned to their respective reference genomes after filtering on read length and removing reads aligning to ambiguous nucleotides (Figure 1A). Our initial analysis using CPD-containing XR-seq reads derived from UV-irradiated human fibroblasts after 1 hour of repair (31) revealed that ∼11% of aligned reads contained exactly one mismatch. The remaining 89% of reads with >1 or 0 mismatches were set aside so that these 11% could be analyzed more closely to determine if they contained deaminated cytosines from CPDs. 70% of these single-mismatch reads contained C > T mismatches (∼8% of aligned reads; Figure 1B), and the vast majority (99%) of these C > T mismatches were in a dipyrimidine sequence context (Figure 1C). Notably, the three cytosine-containing dipyrimidine contexts (CC, CT, and TC) were not equally represented, with CC being almost entirely absent (Figure 1C). C > T mismatches within XR-seq reads peaked between positions 4–10 nucleotides from the 3′ end of the read with very few mismatches observed outside this range, especially near the 5′ end of the read (Figure 1D). This coincides with the expected location of CPD lesions in human excised fragments, which have been reported to be located 5–8 nucleotides from the 3′ ends of XR-seq reads (16,18). Similar C > T mismatch patterns were observed in later repair timepoints (Supplementary Figure S1). In contrast, the distribution of non-C > T mismatches did not show the same enrichment 4–10 nucleotides from the 3′ end of the read in the 1-hour repair timepoint. (Supplementary Figure S2) These results suggest that the C > T mismatches in aligned XR-seq reads represent deamination events in cytosine-containing CPDs.

As a control, we performed a similar analysis with lesions that are not expected to induce cytosine deamination. In published human XR-seq data for 6–4PPs (31) and cisplatin adducts (29), C > T mismatches were no longer dominant, comprising only 15% of total mismatches for 6–4PPs (Supplementary Figure S3A) and 12% for cisplatin adducts (Supplementary Figure S3B). Of those C > T mismatches in 6–4PP and cisplatin XR-seq reads, 79% (Supplementary Figure S3C) and 71% (Supplementary Figure S3D), respectively, were in dipyrimidine contexts, similar to the frequency expected by random chance (75%; i.e. the probability that at least one of the flanking positions is a pyrimidine). The positions for C > T mismatches in 6–4PP-containing reads had a minor peak spanning positions 7–9 nucleotides from the 3′ end, but this signal is largely overshadowed by significant noise at the majority of other positions (Supplementary Figure S3E). Instead of a peak, positioning data for C > T mismatches in cisplatin XR-seq reads display a valley spanning positions 6–8 nucleotides from the 3′ end, likely due to the enrichment of guanine adducts at these positions (29) (Supplementary Figure S3F).

A similar signature of C > T mismatches in CPD-containing XR-seq reads was observed in published data from other species (i.e. *Arabidopsis* (30) and yeast (27); Supplementary Figure S4), as well as TC-NER- and GG-NER-deficient human cells (16) (Supplementary Figure S5). In all cases, C > T mismatches were much more common than other mismatch types, were found almost exclusively in dipyrimidine contexts, and were enriched at the expected positions near the 3′ ends of reads. Taken together, these data indicate that the observed C > T mismatches likely arose from deaminated CPDs, regardless of species or repair background, supporting the use of C > T mismatches as a proxy for CPD locations within XR-seq reads.

### Adapter-specific mispriming results in truncation of a subset of XR-seq reads

**Figure 2.**
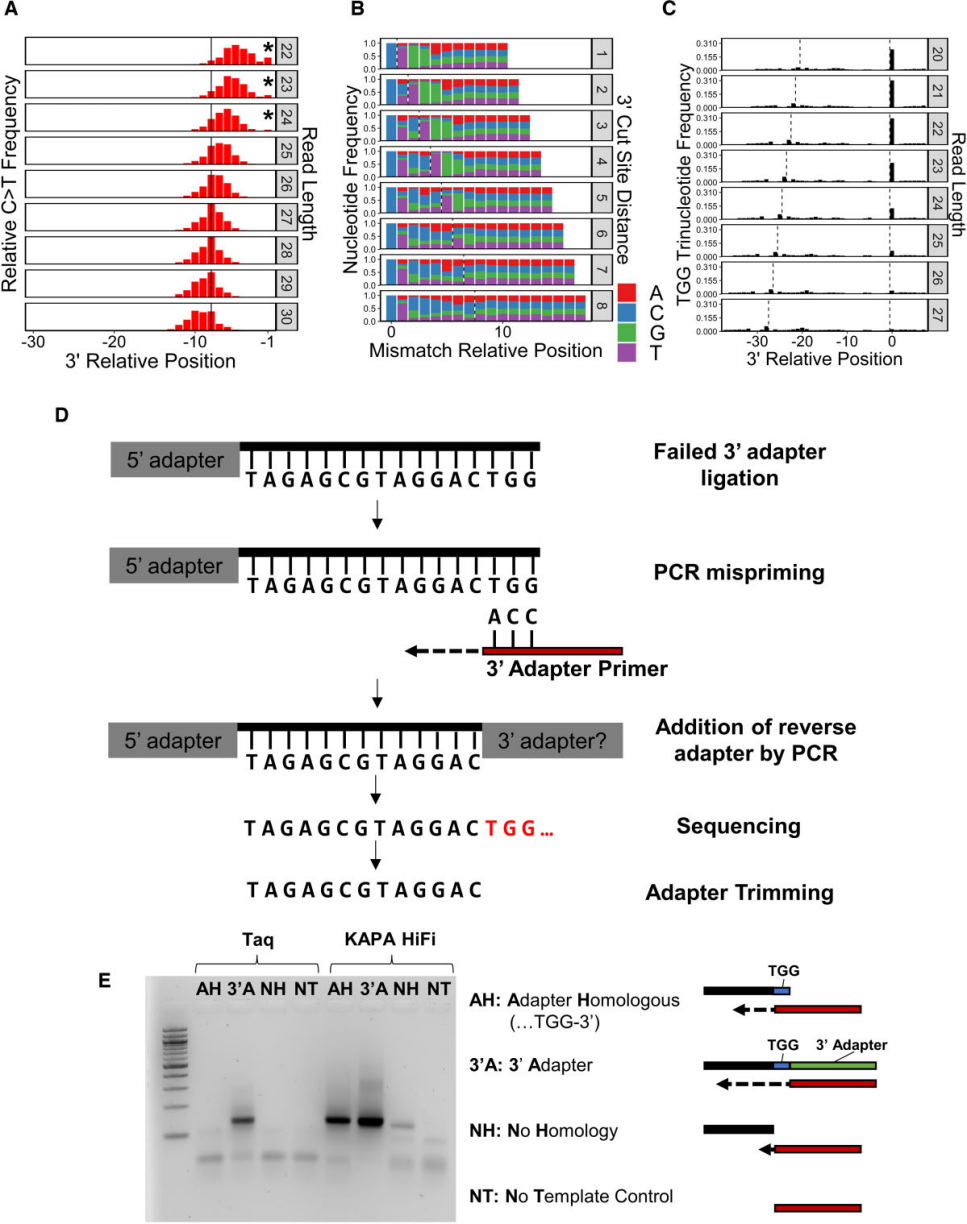
Mispriming due to homology between the 3′ adapter sequence and the 3′ end of a read can result in truncated XR-seq reads. (**A**) Relative frequencies of the occurrence of C > T mismatches at read positions relative to the 3′ end, stratified by read length. Bars in red represent significantly upregulated positions (greater than four standard deviations above the mean of a background sample of the ten positions closest to the 5′ end; *P*< 6.3e-5). The solid line indicates the most frequent mismatch position across all reads. Asterisks indicate positions that clearly stray from the relatively normal distribution of mismatch positions. Position –1 on the x-axis indicates the first nucleotide before the 3′ cut site of the XR-seq read. (**B**) Nucleotide frequencies relative to a 5′-anchored C > T mismatch, stratified by the distance from the mismatch to the perceived 3′ NER cut site. The dashed line represents the 3′ NER cut site, as determined by the 3′ read end after adapter trimming. The 0 position on the x-axis represents the C > T mismatch. (**C**) Frequency of TGG sequences in all reads, regardless of mismatches, stratified by read length. Dashed lines represent the 5′ and 3′ NER cut sites, as determined by the ends of reads after adapter trimming. Positions are relative to the thymine in the TGG sequence. Position –1 on the x-axis indicates the first nucleotide before the 3′ cut site of the XR-seq read. Data were derived from aligned CPD XR-seq reads from NHF1 cells after 1 h of repair (A-C). (**D**) Schematic of the mispriming process which leads to bioinformatic trimming of the original repair fragment sequence. (**E**) Gel showing mispriming from KAPA HiFi polymerase. The first four lanes after the ladder show the results of PCR using EconoTaq polymerase, and the last four lanes show the results of PCR using KAPA HiFi polymerase. The lanes within each set of PCR reactions are differentiated by templates with adapter sequences of varying completeness, further described in the legend to the right of the gel.

Closer inspection of C > T mismatch positions in human CPD XR-seq reads revealed a surprising abundance of mismatches 1–3 nucleotides from the 3′ incision site, (i.e. positions –1 to –3; Figure 1D). This enrichment of 3′ incision sites so near the presumed CPD location is unexpected based on incision patterns established by previous biochemical studies (18). Stratifying the distribution of mismatch positions by read length indicated that this pattern was specific to shorter reads (22–24 nucleotides long; Figure 2A). To further investigate this phenomenon, we examined nucleotide frequencies beyond the 3′ incision site by retrieving the genomic sequence context outside the aligned positions. In doing so, we observed a clear TGG sequence preference immediately 3′ of the incision site, especially for reads with a short cut-site distance (i.e. where the C > T mismatch is < 5 nucleotides from the incision site, inclusive of the mismatch; Figure 2B). Notably, because cut-site distance is directly related to read length, these reads tended to be shorter overall. As a result, we wondered whether this TGG sequence pattern was adjacent to all short XR-seq reads, not just the ∼8% containing C > T mismatches. To address this question, we analyzed the frequency of TGG motifs throughout all XR-seq reads and flanking sequences, stratified by read length. This revealed a significant enrichment of TGG sequences immediately beyond the 3′ incision site in shorter reads (i.e. 20–24 nucleotides). For example, 30% of reads 22 nucleotides in length contain a TGG sequence immediately beyond the 3′ incision site (∼21-fold higher than expected by random chance; Figure 2C). We also observed higher TGG frequencies at the same location in shorter *Arabidopsis* and yeast reads, albeit to a lesser extent (up to ∼8% in *Arabidopsis* and ∼9% in yeast) (Supplementary Figure S6).

This TGG sequence matches the beginning of the adapter that is ligated to the 3′ end of the excised NER fragment during the XR-seq protocol (16,26). Since both 3′ and 5′ adapters must be ligated to the excised NER fragment in order for it to be sequenced, singly-ligated fragments (either 5′ or 3′) would not normally be included in the resulting library. However, we hypothesized that fragments lacking a 3′ adapter could be rescued by PCR mispriming if the 3′ end of the read contained a sequence homologous to the 3′ adapter (e.g. TGG). This hypothesis could potentially explain the abundance of shorter reads with C > T mismatches unexpectedly close to the 3′ incision site, since *in silico* adapter trimming would truncate the 3′ end (Figure 2D).

To test this hypothesis, we performed PCR reactions using a template that mimicked an XR-seq read lacking a 3′ adapter but containing a homologous TGG sequence at the 3′ end. Attempting to amplify this template with a standard Taq polymerase (i.e. EconoTaq) did not produce any appreciable PCR product, but a product was observed for a control template, which contained a full-length 3′ adapter (Figure 2E, Taq lanes AH and 3′A). In contrast, using Kapa HiFi polymerase, as described in the XR-seq protocol (26), resulted in a clear PCR product for the homologous TGG template, which was only slightly less intense than the product produced by the control template with the 3′ adapter (Figure 2E, KAPA HiFi lanes AH and 3′A). Negative controls with either no template sequence or a template sequence without the homologous TGG sequence produced no products in most cases and a very minor product in the case of Kapa HiFi polymerase with the non-homologous template (Figure 2E, lanes NH and NT). These data suggest that the high-fidelity Kapa polymerase is capable of initiating from partially annealed primers (i.e. primer annealed to the homologous 3′ TGG sequence) and may be responsible for the XR-seq mispriming events leading to truncated read sequences.

To avoid bias in subsequent analyses, we removed XR-seq reads with a TGG sequence immediately beyond the 3′ incision site, as our data suggest that these reads are most likely truncated due to mispriming events. This filtering substantially reduced the number of anomalous reads with short 3′ incision site distances at shorter read lengths (Compare figure 2A to Supplementary figure S7A). However, we noticed that sequences beyond the 3′ incision site of shorter reads were still enriched in TG sequences, implying that even this shorter sequence could still cause mispriming and read truncation (Supplementary Figure S7B). Filtering on this TG sequence instead of TGG slightly reduced the number of anomalous short reads as well as the sequence bias following the 3′ incision site (Supplementary Figure S7C, D). However, the marginal returns of this more stringent filtering did not justify the potential bias from removing a much larger proportion of reads. For this reason, all subsequent analyses used the TGG-filtered reads.

### Variability in incision site selection is species-specific

**Figure 3.**
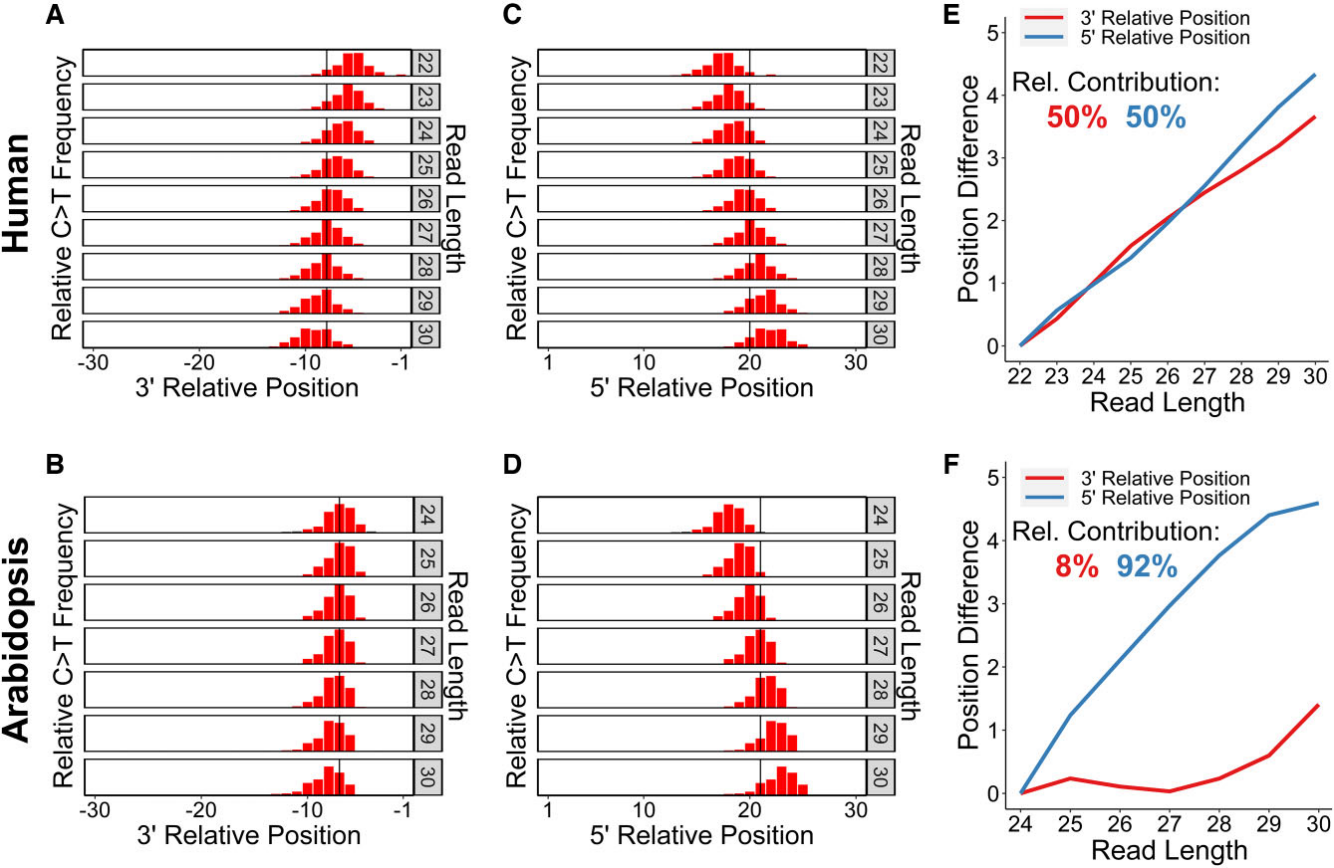
The distance between the lesion and 3′ incision site is more variable in humans than *Arabidopsis*. (**A–D**) Relative frequencies of the occurrence of C > T mismatches at read positions relative to the 3′ (A, B) and 5′ (C, D) cut sites, stratified by read length. Bars in red represent significantly upregulated positions (greater than four standard deviations above the mean of a background sample of the 10 positions closest to the 5′ end; *P*< 6.3e-5). The solid line indicates the most frequent mismatch position across all reads. Positions –1 or 1 on the x-axes indicate the first nucleotide before (A, B) or after (C, D) the related NER cut site, respectively. (**E**, **F**) Relative 5′ and 3′ contributions to read length variability. Position difference is calculated from the mean C > T mismatch position for a given read length and the mean for the shortest read length. Rel. (Relative) contribution is calculated using the weighted sum of the position difference values where weights are determined by the relative frequencies of each read length. Data were derived from CPD XR-seq reads from NHF1 (human) cells after 1h repair (A, C, E) and *Arabidopsis* after 30min repair (B, D, F). For all data, reads with a flanking 3′ TGG sequence were filtered out.

Next, we used C > T mismatches in published CPD XR-seq reads (27,30,31) to investigate the locations of 5′ and 3′ incision sites relative to lesion location, stratified by read length. In human cells, the average distance between the lesion (i.e. C > T mismatch) and the 3′ incision site increased by ∼3.7 nucleotides between the shortest and longest read lengths analyzed (22 and 30 nucleotides, respectively), leading to a clear shift in the distribution of lesion positions relative to the 3′ incision site across different read lengths (Figure 3A). In contrast, *Arabidopsis* displayed relatively little change in the distribution of lesion positions relative to the 3′ incision site across different read lengths (Figure 3B), indicating that the 3′ incision site is largely fixed, regardless of fragment length. Complementary analysis of 5′ incision sites in human XR-seq reads indicated that the distance between the lesion and the 5′ incision site increased by ∼4.3 nucleotides between the shortest and longest fragment lengths (Figure 3C), similar to the variability in the 3′ end. An even more dramatic shift in the 5′ incision site relative to the lesion with increasing read length was observed in *Arabidopsis* (Figure 3D), in contrast to the relatively fixed location of the 3′ incision site.

These patterns are not uniform across the entire range of observed read lengths, and in *Arabidopsis*, the shift in the 5′ incision site relative to the lesion is most prominent at read lengths around the median, which are the most abundant in the XR-seq data set. A more sophisticated representation of the relative contributions of 5′ and 3′ incision site selection to read length variability can be derived by considering these relative read length frequencies. This analysis reveals that in human cells, read length variability is impacted similarly by both incision sites, with ∼50% of variability explained by both the relative positions of 3′ and 5′ incision sites (Figure 3E). These results are consistent in repair deficient backgrounds as well, with variability in the 3′ incision site explaining ∼56% and ∼52% of read length variability in TC-NER- and GG-NER-deficient cells, respectively (Supplementary Figure S8). In *Arabidopsis*, however, the 5′ incision site contributes disproportionately to read length variability (∼92%), while the 3′ incision site contributes very little (∼8%; Figure 3F). In yeast, the 3′ incision site contributes less to read variability than the 5′ incision site (31%, compared to 69%; Supplementary Figure S9), albeit over a much smaller range of read lengths analyzed (22–25 nucleotides). These patterns highlight species-specific differences in 5′ and 3′ incision site selection relative to CPD lesions during NER.

### 5′ NER incision sites exhibit sequence specificity in eukaryotes

**Figure 4.**
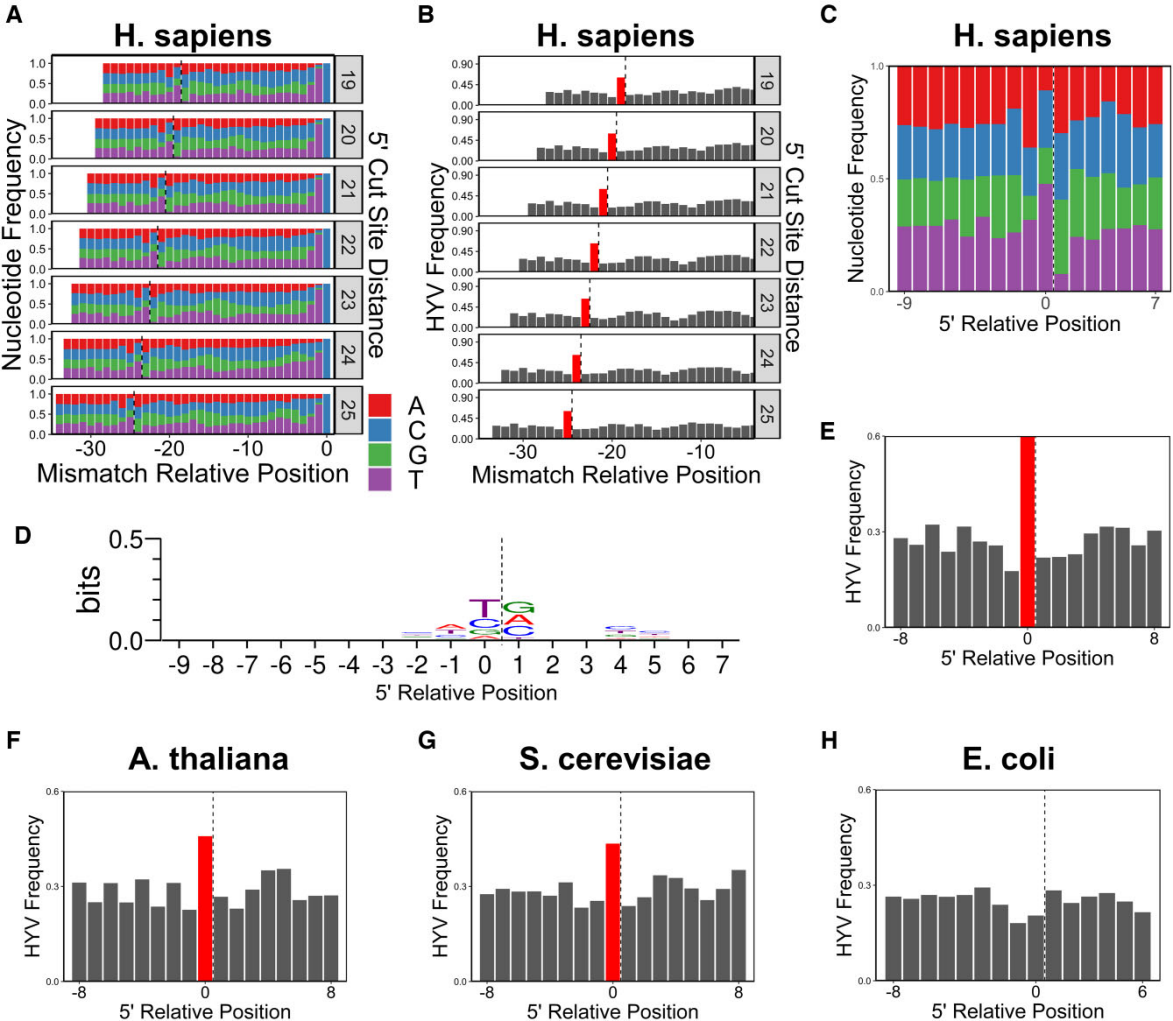
The 5′ incision site occurs preferentially at HYV sequences in eukaryotes. For plots where the x-axis is relative to the 5′ end (C, E–H), the 1 position indicates the first nucleotide after the 5′ NER cut site. (**A**) Nucleotide frequencies relative to a 3′-anchored C > T mismatch, stratified by the distance from the mismatch to the 5′ NER cut site. The dashed line represents the 5′ NER cut site. The 0 position on the x-axis indicates the C > T mismatch. (**B**) Relative HYV sequence frequency in reads with C > T mismatches, stratified by the distance from the mismatch to the 5′ NER cut site. The dashed line represents the 5′ NER cut site. Positions are relative to the pyrimidine in the HYV sequence. Bars in red represent significantly upregulated positions (greater than four standard deviations above the mean of a background sample of the six 5′-most positions; *P*< 6.3e-5). The 0 position on the x-axis indicates the C > T mismatch. Positions close to the mismatch are omitted due to nucleotide bias resulting from CPD-forming sequences. (**C**) Nucleotide frequency data, similar to plot A but calculated across all reads regardless of mismatches. (**D**) Sequence logo calculated from the data represented in plot C. The information content is normalized to the GC content of input read sequences. Note that the y-axis is shortened from the maximum information content of 2 bits in order to emphasize the relevant patterns. (**E–H**) HYV sequence frequency data, similar to plot B but calculated across all reads, regardless of mismatches. Position 1 on the x-axis indicates the first nucleotide after the 5′ NER cut site (C-H). Data were derived from CPD XR-seq reads from NHF1 (human) cells after 1h repair (A–E), *Arabidopsis* after 30min repair (F), yeast after 20 min repair (G) and *E. coli* after 5 min repair (H). For all data, reads with a flanking 3′ TGG sequence were filtered out.

In order to better understand the mechanisms contributing to NER incision patterns, we looked at nucleotide frequencies flanking the 5′ end of published human CPD XR-seq reads (31), similar to our previous analysis at the 3′ end (Figure 2B). These data revealed a depletion of guanine two nucleotides upstream of the 5′ incision site, an enrichment of pyrimidines (especially thymine) one nucleotide upstream of the incision site, and a depletion of thymine one nucleotide downstream of the incision site (Figure 4A). These sequence preferences yield an HYV (not G; C/T; not T) pattern around the 5′ incision site of reads, with the incision situated between the ‘Y’ and ‘V’ positions. Unlike the TGG sequence bias flanking a subset of 3′ incision sites, this sequence preference is unlikely to be due to PCR mispriming or other similar experimental artifacts, because the HYV sequence is much less specific, extends partially into the read sequence itself, and is not homologous or complementary to the 5′ primer. Furthermore, this pattern is present regardless of the distance between the incision site and the mismatch (i.e. the lesion) with HYV frequencies ranging from 60–63%, all of which are highly significant (*P* < 6.3e-5; Figure 4B). Given this independence from the distance between the lesion and the 5′ incision site, we extended our analysis to all aligned XR-seq fragments, regardless of whether or not a C > T mismatch was present. The resulting nucleotide frequencies are consistent with the HYV pattern, with a depletion of guanine two nucleotides upstream of the 5′ incision site (∼11% of bases), an enrichment of pyrimidines one nucleotide upstream of the incision site (∼73%), and a depletion of thymine one nucleotide downstream of the incision site (∼8%; Figure 4C,D). Calculating HYV frequencies across these combined data confirmed that the pattern is significantly enriched in human XR-seq reads at the 5′ incision site (∼60%; *P* < 6.3e-5) (Figure 4E).

Next, we tested whether this HYV sequence pattern was enriched at the 5′ NER incision site in published XR-seq reads derived from other species (27,30). In *Arabidopsis* and yeast, the HYV pattern was significantly enriched flanking the 5′ incision site, with the incision site between the ‘Y’ and ‘V’ nucleotides of the pattern (∼46% and ∼44% respectively; *P* < 6.3e-5; Figure 4F, G). In contrast, there was no enrichment of HYV sequences at the 5′ incision site in published XR-seq reads derived from the prokaryote, *Escherichia coli* (54). In fact, the HYV sequence was significantly depleted at this position (frequency of 20%; *P* < 6.3e-5; Figure 4H). The HYV pattern is not restricted to a particular lesion or repair pathway as it is significantly enriched at the 5′ incision site in human reads containing 6–4PPs and cisplatin lesions and in TC-NER- and GG-NER-deficient systems (Supplementary Figure S10). We performed a similar analysis of the 3′ incision site after filtering on ‘TGG’ sequences, but there was no consistent sequence pattern across the three analyzed eukaryotes (Supplementary Figure S11). In summary, these findings suggest that in eukaryotic cells, the 5′ NER incision reaction occurs preferentially in sequences that match an HYV pattern.

### A short-read alignment algorithm maps XR-seq reads containing tandem CC to TT deamination events

Cytosine bases in a CPD with a CC sequence can tandemly deaminate, resulting in two adjacent C > T mismatches (CC > TT). However, our analysis indicates these tandem mismatches are largely absent in aligned CPD XR-seq reads (835 reads in ∼50 000 000 from the human CPD XR-seq data at the 1-h repair timepoint), even though our published CPD-seq data indicate CC CPDs comprise ∼10% of all CPD lesions (compared to ∼53% TT CPDs) (35,55). We hypothesized that because XR-seq reads are relatively short (usually <30 nucleotides), the two adjacent mismatches resulting from a tandem deamination event might preclude alignment by standard short read alignment algorithms, such as Bowtie2 (56). Bowtie2 does not allow multiple mismatches during seeding, which is a crucial step during the early stages of the alignment process. Accordingly, a short read with multiple mismatches may not contain any seed sequence of appreciable length, preventing it from aligning.

**Figure 5.**
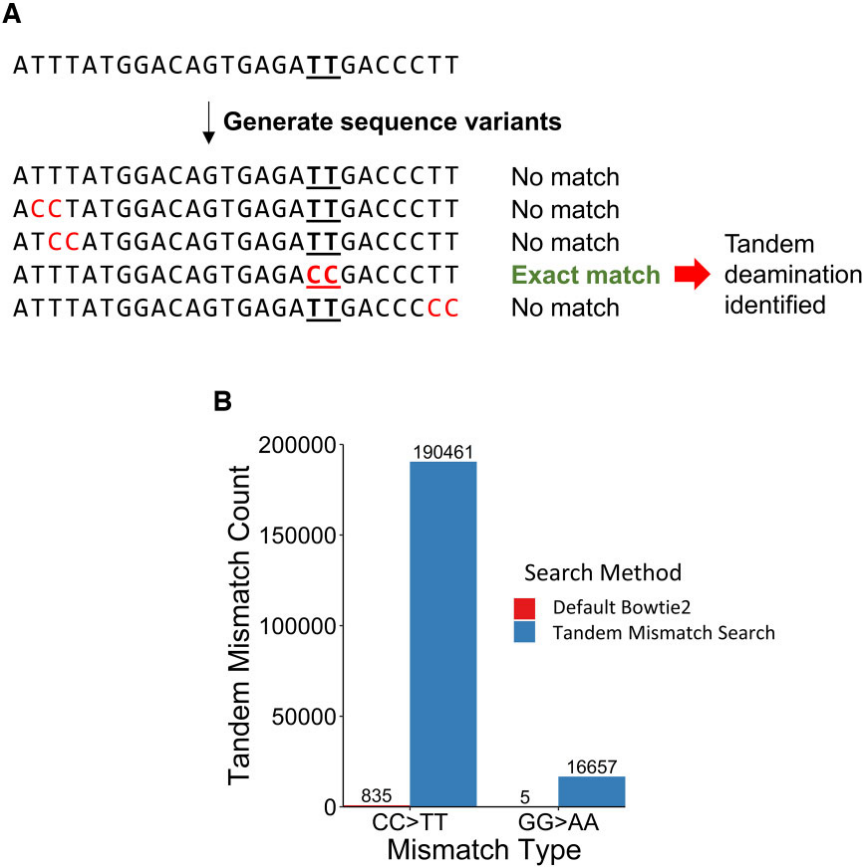
A short read alignment protocol aligns tandemly deaminated CPD XR-seq reads. (**A**) Schematic for the tandem alignment protocol. Underlined bases represent the true tandem deamination event. Paired red bases represent hypothetical tandem deamination events. (**B**) Counts of identified tandem mismatches in aligned NHF1 CPD XR-seq reads after 1 h of repair using a default bowtie2 alignment or the specialized alignment protocol shown above (A). These counts are prior to filtering based on read length or 3′ TGG sequences.

To overcome these limitations, we designed an algorithm to align XR-seq reads containing CC > TT mismatches. This algorithm generates sequence variants by individually replacing each TT dinucleotide with CC, potentially reversing a tandem deamination event (Figure 5A). To test if such a reversal has occurred, exact matching between sequence variants and the reference genome is performed using Bowtie2. If only one variant returns an exact match and the original read sequence does not match, an alignment is recorded with a tandem mismatch at the dinucleotide that distinguished the variant (Figure 5A). Compared to default alignment parameters with Bowtie2, this algorithm produced over 200-fold more alignments containing CC > TT mismatches (Figure 5B). Furthermore, reads with CC > TT mismatches were over ten times as common as those with a control GG > AA mismatch sequence (Figure 5B). Notably, these CC > TT mismatches were much less common overall than single C > T mismatches (0.3% of reads compared to 7.8% for single C > T mismatches at the 1 hour repair timepoint), but the relative proportion of CC > TT mismatches compared to C > T mismatches increased at later timepoints, possibly owing to more complete deamination throughout the genome (Table S2).

**Figure 6.**
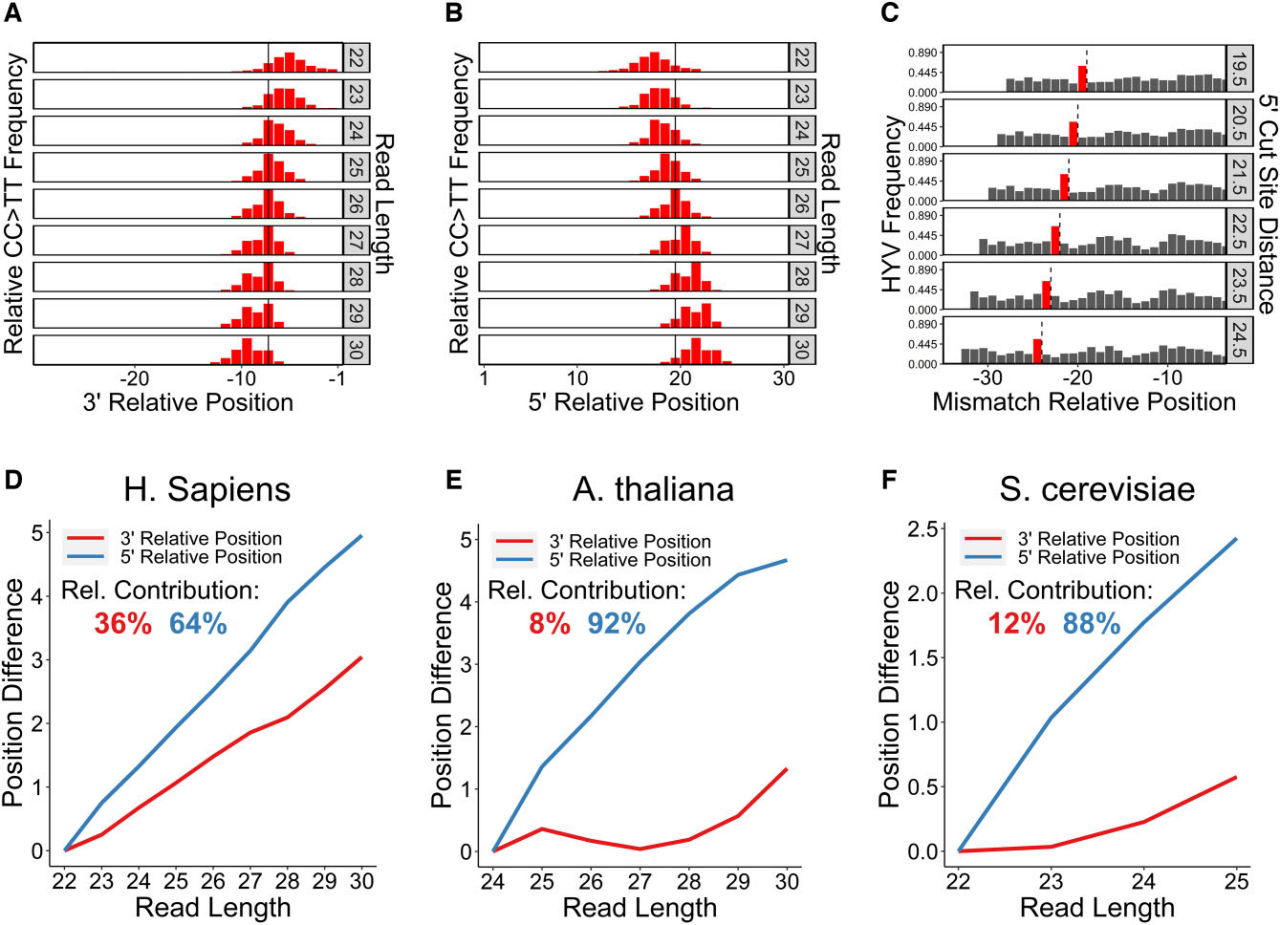
Tandem deamination data follow patterns indicative of CPD positioning and reveal similar HYV cut site preference and species-specific incision variability. (A, B) Relative frequencies of the occurrence of CC > TT mismatches at read positions relative to the 3′ (**A**) and 5′ (**B**) cut sites, stratified by read length. Bars in red represent significantly upregulated positions (greater than four standard deviations above the mean of a background sample of the 10 positions closest to the 5′ end; *P*< 6.3e-5). The solid line indicates the most frequent mismatch position across all reads. Positions –1 or 1 on the x-axes indicate the first nucleotide before (A) or after (B) the related NER cut site, respectively. (**C**) Relative HYV sequence frequency in reads with CC > TT mismatches, stratified by the distance from the mismatch to the 5′ NER cut site. The dashed line represents the 5′ NER cut site. Positions are relative to the pyrimidine in the HYV sequence. Bars in red represent significantly upregulated positions (greater than four standard deviations above the mean of a background sample of the six 5′-most positions; *P*< 6.3e-5). On the x-axis, the 0 position represents the half-base position between the bases in the CC > TT tandem mismatch. Positions close to the mismatch are omitted due to nucleotide bias resulting from CPD-forming sequences. (**D–F**) Relative 5′ and 3′ contributions to read length variability. Position difference is calculated from the mean C > T mismatch position for a given read length and the mean for the shortest read length. Rel. (Relative) contribution is calculated using the weighted sum of the position difference values where weights are determined by the relative frequencies of each read length. Data were derived from CPD XR-seq reads from NHF1 (human) cells after 1 h repair (A–C), *Arabidopsis* after 30 min repair (D), and yeast after 20min repair (E). For all data, reads with a flanking 3′ TGG sequence were filtered out.

Analysis of CC > TT mismatch locations within published human XR-seq reads (31) indicated that they peak at positions 5–10 nucleotides from the 3′ incision site and 17–22 nucleotides from the 5′ incision site (Figure 6A,B), a pattern that is highly similar to C > T single mismatches (Figure 3A, C) and coincides with known CPD lesion locations in excised NER fragments (16,18). In contrast, control GG > AA mismatches are not enriched at these positions but instead are widely distributed throughout XR-seq reads (Supplementary Figure S12). Taken together, these results indicate that CC > TT mismatches likely indicate the location of tandemly deaminated CPDs within XR-seq reads.

Additionally, we investigated incision patterns relative to the CC > TT mismatches in published XR-seq data (27,30,31). Human XR-seq reads containing these mismatches have a significant enrichment of the HYV sequence pattern around the 5′ incision site, independent of the distance between the incision site and the mismatches (56–65%; *P* < 6.3e-5; Figure 6C). Investigation of the distance between CC > TT mismatches and the 5′ and 3′ incision sites revealed that 3′ incision site selection contributes more to fragment length variability in humans than in *Arabidopsis* and yeast (∼36% of variability in humans compared to ∼8% and ∼12% in *Arabidopsis* and yeast, respectively; Figure 6D–F). These data show the same relative trend as the single C > T mismatch data, with greater 3′ contributions to variation in fragment length in humans compared to *Arabidopsis* and yeast, although the calculated percentages in humans and yeast were somewhat lower for single C > T mismatches. Taken together, these findings indicate that while a significant number of CPD XR-seq reads are normally not aligned by standard short read alignment algorithms due to the presence of tandem cytosine deamination events at CC CPD lesions, these can be efficiently aligned using the custom algorithm described herein.

### 5′ and 3′ incision site locations are coupled

**Figure 7.**
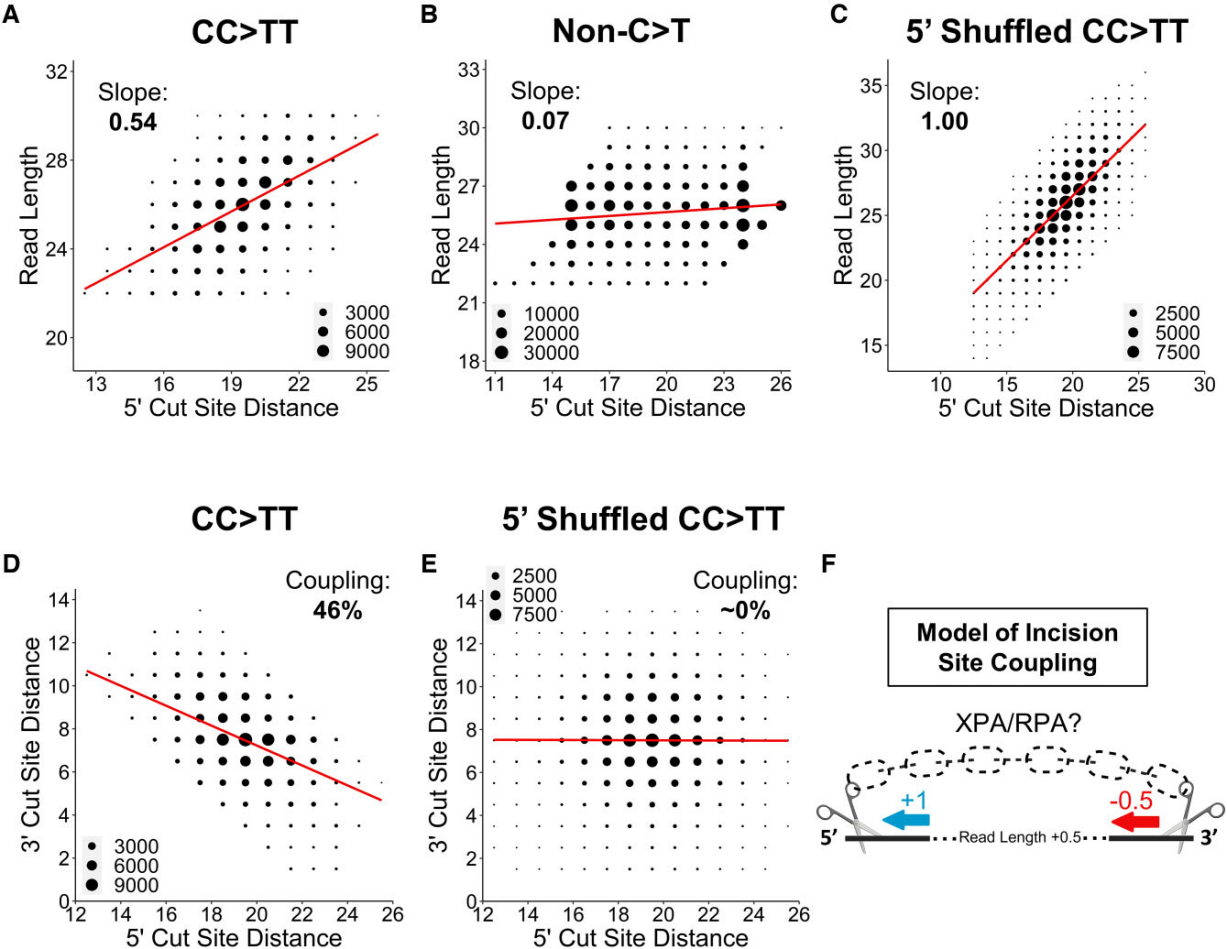
5′ incision site selection influences 3′ incision site selection. (A–F) Counts of read lengths (A–C) or 3′ NER cut site distances (D, E) for each 5′ NER cut site distance. Cut site distance was determined using either CC > TT mismatch location as a proxy for CPD location (A, D), single non-C > T mismatches, where the mismatch is treated as an NER lesion (**B**), or a shuffled distribution of the 5′ cut site distances derived from CC > TT mismatches (C derived from A). Cut site distance is inclusive of the half-base position between the bases in the CC > TT mismatch (A, **C–E**) or the base in the single mismatch (B). The size of each point on the plot represents the number of XR-seq reads associated with it, as described by the corresponding legend. The red lines represent the linear regression for the data. Coupling percentages were calculated by multiplying the slope of the linear regression by -100% (D, E). Data were derived from CPD XR-seq reads from NHF1 (human) cells after 1 h repair. For all data, reads with a flanking 3′ TGG sequence were filtered out. (**F**) Graphical representation of incision site coupling. The model visualizes incision patterns in human NER (A, D). The blue arrow represents changes in the 5′ cut site distance, and the red arrow represents corresponding changes in the 3′ cut site distance. The dashed chain represents partial coupling between the incision events. Created with biorender.com.

Since our data indicate a sequence preference for the 5′ incision by XPF-ERCC1 (Figure 4), which is known to occur first in the NER reaction (15,25), we wondered to what extent the location of the 5′ incision event affects overall fragment length. To address this question, we compared 5′ incision site distance and fragment length in published human CPD XR-seq reads (31) containing tandem CC > TT mismatches, since these tandem mismatches precisely specify the location of the CPD lesion. This analysis revealed a significant correlation between 5′ incision site distance and fragment length (*P*< 1e-15), and the corresponding regression lines had slopes of 0.54 and 0.55, for the 1 and 8 h repair timepoints, respectively (Figure 7A and S13A). In other words, a 1 nucleotide increase in 5′ incision site distance from the lesion was associated with an average ∼0.55 nucleotide increase in fragment length (Figure 7A and S13A). A similar analysis of single C > T mismatches in CPD XR-seq reads also revealed a significant correlation between 5′ incision site distance and fragment length with slopes of 0.45 and 0.44 for the 1h and 8h repair timepoints, respectively (Supplementary Figure S14A,B). The slightly lower slope observed in the C > T mismatch data may reflect less precise lesion location compared to the tandem mismatch data (i.e. a single C > T mismatch could represent either the 5′ or 3′ base in a CPD). As a control, we performed a similar analysis on the inferred 5′ incision distance from non-C > T mismatches in human CPD XR-seq reads at the 1h repair timepoint. The correlation between the inferred 5′ incision site distance and read length was much lower in this analysis, with a slope of 0.07 (Figure 7B), even though fragments containing these non-C > T mismatches were still subject to the same read length and position filtering constraints as reads with C > T mismatches. Taken together, these findings indicate that increased 5′ incision distance is associated with longer excision fragments during the repair of CPD lesions in human cells.

Our analysis so far indicates that a 1 nucleotide increase in 5′ incision distance relative to the CPD lesion during NER is associated with an ∼0.5 nucleotide increase in fragment length in human cells. In contrast, shuffling 5′ incision site distances to simulate independent 5′ and 3′ incision events yielded a regression line with a slope of 1.00 (i.e. a 1 nucleotide increase in 5′ incision site distance from the shuffled control was associated with an average 1.00 nucleotide increase in fragment length; Figure 7C). This analysis indicates that in human NER, increases in 5′ incision site distance may be partially compensated by a decrease in 3′ incision site distance. To test this hypothesis, we directly compared 5′ and 3′ incision site distances in human CPD XR-seq reads containing tandem CC > TT mismatches. This analysis revealed that the 5′ and 3′ incision site distances were negatively correlated (*P*< 1e-15), and the corresponding regression lines had slopes of –0.46 and –0.45 for the 1 and 8 h repair timepoints, respectively (Figures 7D and S13B). In other words, a 1 nucleotide increase in 5′ incision site distance results in an average ∼0.45 nucleotide decrease in 3′ incision site distance (Figures 7D and S13B). We converted this slope to a ‘coupling coefficient’ (defined as slope multiplied by –100%) to quantify how responsive the 3′ incision site distance is to changes in the 5′ incision site (i.e. how coupled the two incision sites are). This new metric yields a coupling coefficient of 46% for human XR-seq reads containing CC > TT mismatches (Figure 7D) and a roughly similar value (∼55%; Supplementary Figure S14C, D) for human XR-seq reads containing a single C > T mismatch. In contrast, similar analysis of the shuffled CC > TT control, in which the 5′ and 3′ incision site distances are each derived from different NER fragments, yielded 0% coupling (Figure 7E). We also used published CPD XR-seq data from *Arabidopsis* (30) and yeast (27) to investigate incision site coupling in other eukaryotes. Both species exhibited partial coupling of the 5′ and 3′ incision sites, similar to what was observed in human XR-seq data, although the values of the coupling coefficients varied to some extent (Supplementary Figure S15). Taken together, these results indicate that in eukaryotic NER, the location of the 3′ incision site is dependent in part on the distance of the 5′ incision from the lesion, suggesting that the two incision events are partially coupled (Figure 7F).

### NER incision patterns show significant variation at nucleosome boundaries

**Figure 8.**
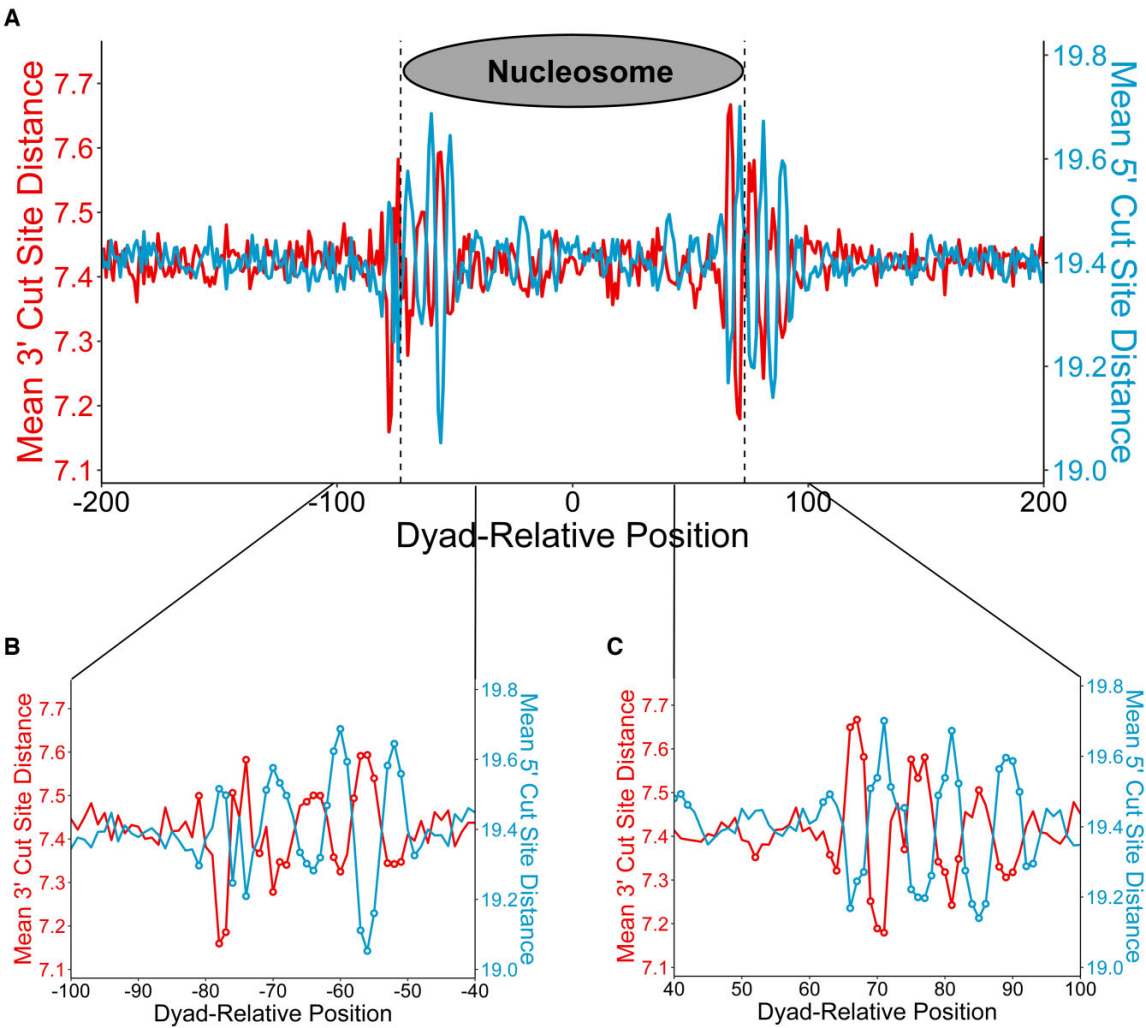
Lesions located near the edges of nucleosomes exhibit greater variability in incision site selection. (**A**) Mean NER cut site distances for lesions at positions relative to nucleosome dyads. (**B**, **C**) Highlighted regions from plot A. Cut site distance was derived from single-C > T mismatches treated as CPD lesions and is inclusive of the mismatch position. Dashed lines represent the nucleosome boundaries at positions –73 and 73 (A). Open circles represent positions where the respective cut site distance is significantly above or below the mean (*P* < 5e-5; B, C). Data is strand-aligned so that all 5′ incisions occur in the negative direction and all 3′ incisions occur in the positive direction, relative to the lesion position. Data were derived from CPD XR-seq reads from NHF1 (human) cells after 1 h repair. For all data, reads with a flanking 3′ TGG sequence were filtered out.

While it is known that packaging of DNA into nucleosomes inhibits the initial steps of NER (32,34,57–59), whether nucleosomes affect NER incision events is unclear. To address this question, we analyzed NER incision events adjacent to nucleosome positions. Specifically, 5′ and 3′ incision site distances were derived relative to single C > T mismatches in published CPD XR-seq reads (31) and analyzed using a previously published map of ∼12 million nucleosomes (34,48). The average 5′ and 3′ incision site distances from the CPD lesion (defined by the location of the C > T mismatch) were plotted across all lesions relative to the nucleosome dyads. Overall, there was relatively little variation in the average 5′ and 3′ incision site distances, which were generally 19.4 and 7.4 nucleotides, respectively (Figure 8A). However, these distances showed significantly greater variation in the ∼30 bp regions around the edges of nucleosomes (±0.2 nucleotides for the 3′ mean incision site distance and ±0.3 nucleotides for the 5′ mean incision site difference; *P* < 5e-5; Figure 8). Closer inspection revealed that increases in the average 5′ incision site distance were compensated by decreases in the 3′ incision site distance and vice versa, consistent with coupling of the incision sites (Figure 8B, C). Similar results were obtained for the 8h repair timepoint as well (Supplementary Figure S16). These findings suggest that NER incision patterns are modulated at nucleosome boundaries in human cells.

## Discussion

The NER pathway plays a critical role in repairing helix-distorting lesions by excising short, lesion-containing DNA fragments. However, the molecular mechanisms that regulate the location of NER incision events relative to the lesion are not fully understood. The XR-seq protocol has proven to be a powerful tool for investigating NER activity in cells by sequencing these excised fragments (26), but analysis of incision events using these data are limited because the precise location of the lesion within the excised fragment is unknown. Here, we have shown that the position of UV-induced CPD lesions in XR-seq reads can be inferred from characteristic C > T and CC > TT mismatches in the alignment to the reference genome, which likely arise due to accelerated cytosine deamination. Analysis of CPD positions relative to incision sites revealed that overall variability in excised fragment length is primarily due to 5′ incision site selection in *Arabidopsis* and yeast, while in humans, both incision sites contribute similarly. Investigation of nucleotide sequences surrounding the 5′ incision site revealed a conserved HYV sequence motif in eukaryotes that is attributable to the endonuclease (e.g. XPF-ERCC1) responsible for the 5′ incision. In contrast, sequence preferences around the 3′ incision site were inconsistent across eukaryotes, once reads that were likely truncated due to mispriming events were removed. Instead, our analysis suggests that the location of the 3′ incision site is dependent on the 5′ incision distance, indicating that the two incision events are partially coupled. Finally, analysis of incision events around nucleosomes revealed oscillating patterns in incision site distance at nucleosome boundaries, suggesting that chromatin may affect incision site selection during NER.

Analysis of sequences flanking the 5′ NER incision site revealed a sequence bias in eukaryotes that is characterized by guanine depletion at the second nucleotide 5′ of the incision, pyrimidine enrichment (especially thymine) immediately 5′ of the incision, and thymine depletion immediately 3′ of the incision (i.e. HYV, with the incision site between the Y and V). We hypothesize that the sequence bias at the 5′ incision site likely reflects sequence specificities of the 5′ endonuclease during NER. Previous studies characterizing XPF-ERCC1 incision on model DNA substrates have reported preference for incision immediately following a pyrimidine base (19,21,24), with especially high cleavage efficiency when thymine is at this position and reduced efficiency when guanine is upstream (i.e. 5′) of the incision site (19). These findings are consistent with the first two positions of the HYV sequence pattern. Additionally, the preference for XPF-ERCC1 to cleave 3′ of thymine bases may explain why this base rarely occurs downstream of the incision site. Taken together, our results suggest a model in which the observed variability in 5′ incision distance (relative to the lesion) arises from sequence preferences of the XPF-ERCC1 endonuclease during NER in cells.

In contrast, sequences surrounding the 3′ incision sites of repair fragments had less consistent patterns across eukaryotic species. This agrees with previous findings that the 3′ endonuclease (XPG) is primarily structure-specific and lacks sequence specificity (23,53,60). Moreover, we also observed species-specific differences in the contribution of the 3′ incision to NER fragment length. An alignment of the protein sequences of 3′ endonucleases in humans (XPG), *Arabidopsis* (UVH3), and yeast (Rad2) reveals that while regions which are critical for catalytic function are highly conserved, other regions, such as those proposed to interact with XPD in humans (53), are less similar (Supplementary Figure S17). Given the important role of XPD in creating the single stranded DNA-double stranded DNA (ssDNA-dsDNA) junction that XPG cleaves (11,23,61), these differences hint at a potential explanation for the species-specific differences in 3′ incision.

Notably, we initially observed consistent enrichment of TGG sequences immediately beyond the 3′ incision site, particularly in short XR-seq fragments. However, our bioinformatic analysis and experimental data indicate that this enrichment is likely due to mispriming events in excised fragments lacking a 3′ adapter but ending in an adapter-homologous TGG sequence. As we have shown that mispriming is primarily caused by the Kappa HiFi polymerase, it may be prudent to consider using a different DNA polymerase in future XR-seq experiments. Additionally, in order to avoid potential artifacts, bioinformatic analysis of XR-seq data could benefit from filtering reads with 3′ flanking sequences (i.e. TGG) indicative of mispriming.

While this study did not find evidence for a consistent sequence pattern guiding the 3′ NER incision event, our data indicate that this incision site is instead significantly influenced by the distance of the 5′ incision event relative to the lesion. While it is known that the 5′ incision occurs prior to the 3′ incision during NER (15), to what extent the 5′ incision event influences the subsequent 3′ incision was previously unclear. Our analysis of cellular NER fragments revealed significant coupling between the 5′ and 3′ incisions in humans and other eukaryotes, with more distal 5′ incisions resulting in more lesion-proximal 3′ incisions and vice versa. A previous study suggested that longer single-stranded DNA (i.e. following 5′ incision) promotes incision efficiency by an XPD-XPG complex *in vitro* (53), which could explain the observed coupling in our data. Another possible explanation involves a recent structural model of the NER pre-incision complex, where looping of the lesion-containing ssDNA brings the 5′ and 3′ ssDNA-dsDNA junctions into close proximity (62). In this model, incision site coupling may be driven by more direct communication between the 5′ and 3′ endonucleases, perhaps mediated by the key NER factors XPA and RPA (62) (Figure 7F).

Our analysis indicates that NER incision patterns are also significantly modulated in human chromatin. It is known that nucleosomes significantly impact the initiation of GG-NER (32–34,63), but it was previously unclear how nucleosomes affect incision site selection, which occurs later in the NER pathway. Our data indicate that 5′ and 3′ incision site distance vary in a periodic fashion near nucleosome boundaries. Notably, the 5′ and 3′ incision site distances show significant coupling, with increases in 5′ incision distance partially compensated for by decreases in 3′ incision distance (and vice versa). These incision patterns may reflect structural constraints on the NER reaction imposed by neighboring nucleosomes, or alternatively may arise from sequence biases (e.g. frequency of HYV sequences) in nucleosomal and linker DNA.

In conclusion, these findings offer new insights into the biochemistry of the NER reaction and suggest hypotheses for how it is accommodated in cellular chromatin. Moreover, they highlight the utility of analyzing characteristic C > T (and CC > TT) mismatches to precisely locate lesions in XR-seq reads. These same analysis methods could be used in future studies to specifically measure repair of deaminated CPDs, which are thought to be a primary cause of mutations in human skin cancers (64), although this would require altering the XR-seq protocol (26) to eliminate high temperature steps prior to damage reversal. Additionally, our findings suggest methods for improved processing of XR-seq data, a commonly used method for analysis of NER activity (16,27,29,30,54,65–67), including filtering reads with 3′ flanking sequences (i.e. TGG) indicative of mispriming and a new protocol for aligning XR-seq reads with CC > TT mismatches. Future studies could adapt the bioinformatics methods described herein to analyze repair of other classes of DNA lesions (e.g. benzo[a]pyrene adducts, etc.) at single nucleotide resolution, potentially by employing translesion DNA polymerases in the XR-seq protocol that introduce characteristic mismatches at the location of the lesion.

## Supplementary Material

gkad1195_Supplemental_File

## Data Availability

The XR-seq reads used throughout this analysis can be found through the Gene Expression Omnibus (GEO) database using the following accession IDs and links: *Arabidopsis*: GSE108932 (30), *E. coli*: GSE92734 (54), Human: CSB mutant: GSE67941 (16), GM12878 (cisplatin): GSE82213 (29), NHF1 (UV): GSE76391 (31), XPC mutant: GSE67941 (16), Yeast: GSE110621 (27), The MNase data used to map nucleosome dyads can be found through the GEO database using the following accession ID: GSE36979 (48). The Duke and DAC blacklisted regions used to filter nucleosome dyads can be found in this project's code repository (https://doi.org/10.5281/zenodo.10211636) at perl/DAC_Duke_hg19_excludable.bed. Alternatively, the file can be created manually by merging the regions in the original Duke and DAC excludable bed files which can be obtained from the UCSC genome browser here: https://genome.ucsc.edu/cgi-bin/hgFileUi?db=hg19&g=wgEncodeMapability.
