# Supplementary material for: Analysis of cytosine deamination events in excision repair sequencing reads reveals mechanisms of incision site selection in NER
Source: Nucleic Acids Res. 2023 Dec 18;52(4):1720–35. doi: 10.1093/nar/gkad1195 (PMC10899786; doi:10.1093/nar/gkad1195)
Supplement: gkad1195_Supplemental_File [file gkad1195_supplemental_file.pdf]

SUPPLEMENTAL MATERIALS

Oligo Name	DNA Sequence
Adapter-homologous Template (AH)	**GTTCAGAGTTCTACAGTCCGACGATC**CATTTAGACATGCCTTTGCTTCTCCTTGG
Full 3' Adapter Template (3'A)	**GTTCAGAGTTCTACAGTCCGACGATC**CATTTAGACATGCCTTTGCTTCTCCTTGG**TGGAATTCTCGGGTGCCAAGG**
Non-homologous Template (NH)	**GTTCAGAGTTCTACAGTCCGACGATC**CATTTAGACATGCCTTTGCTTCTCCT
Forward Primer	AATGATACGGCGACCACCGAGATCTACACGTTCAGAGTTCTACAGTCCGA
Reverse Primer	AAGCAGAAGACGGCATACGAGATATGCGAGTGACTGGAGTTCCTTGGCACCCGAGAATTCCA

Table S1: Oligonucleotide sequences used to test for PCR mispriming events. The abbreviations given in the first three rows correspond to Figure 2E. Complete adapter sequences are bolded and underlined. The core read sequence for the templates (i.e., CATTT…CTTGG) was selected from an XR-seq sequence that was suspected of mispriming.

Timepoint	Percentage of Reads with Single C>T Mismatches	Percentage of Reads With CC>TT Mismatches	Ratio of CC>TT to C>T Mismatches
1h	7.83%	0.31%	0.040
8h	4.66%	0.19%	0.040
24h	3.01%	0.13%	0.043
48h	2.85%	0.13%	0.047

Table S2: Single and tandem deamination rates vary over time. Data corresponds to CPD XR-seq reads in NHF1 (human) cells. The timepoint refers to the amount of time cells had to repair damage following UV exposure.

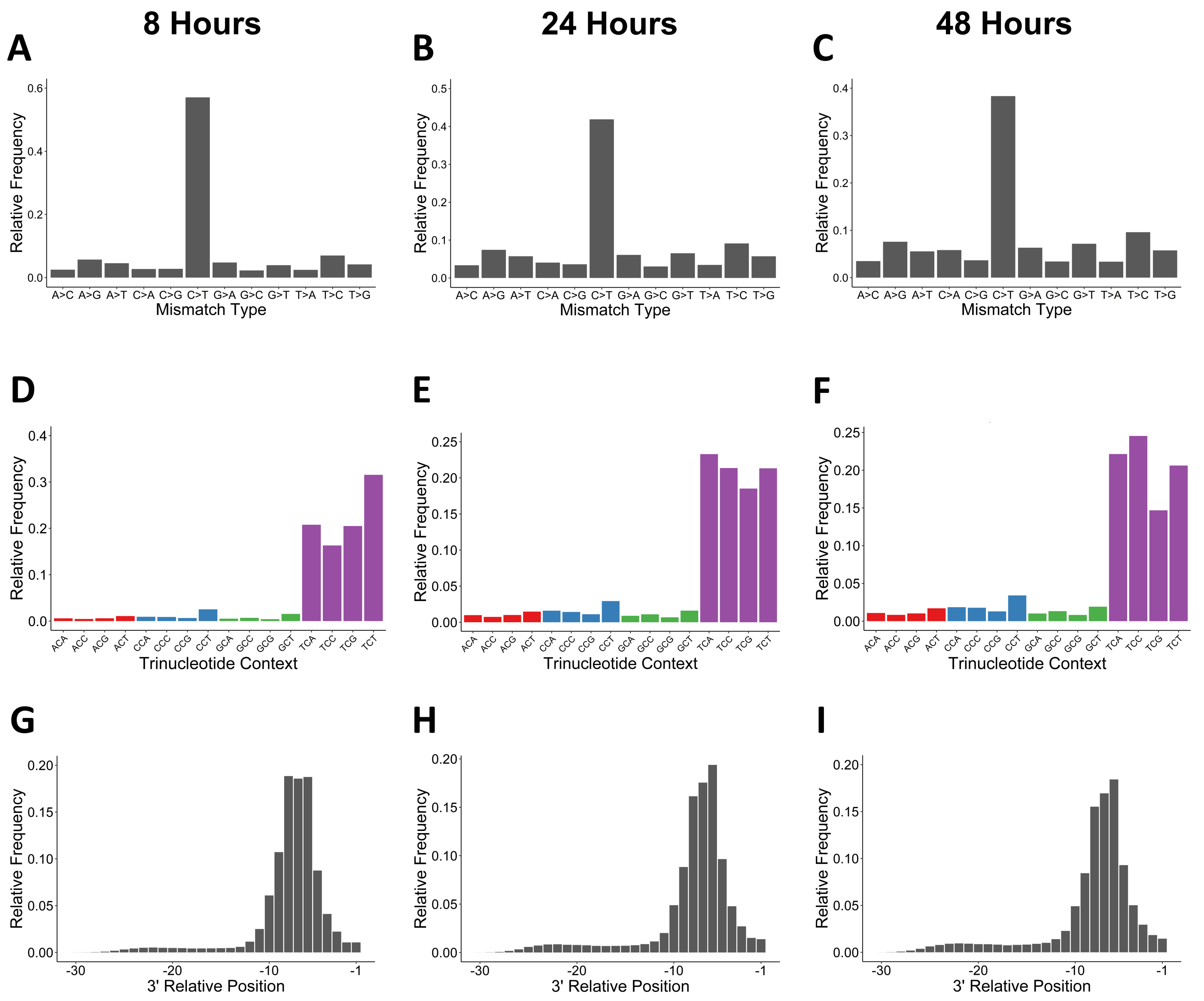

Figure S1: C>T mismatches in aligned XR-seq reads are indicative of CPD positions across different repair timepoints. (**A-C**) Frequencies of each of the 12 single nucleotide mismatch types. (**D-F**) Frequencies of each of the 16 trinucleotide contexts for C>T mismatches. (**G-I**) Frequencies of the occurrence of C>T mismatches at read positions relative to the 3’ end. Position -1 on the x-axes represents the first nucleotide before the 3’ cut site. Data were derived from CPD XR-seq reads from NHF1 cells after 8h (A,D,G), 24h (B,E,H), and 48h (C,F,I) repair.

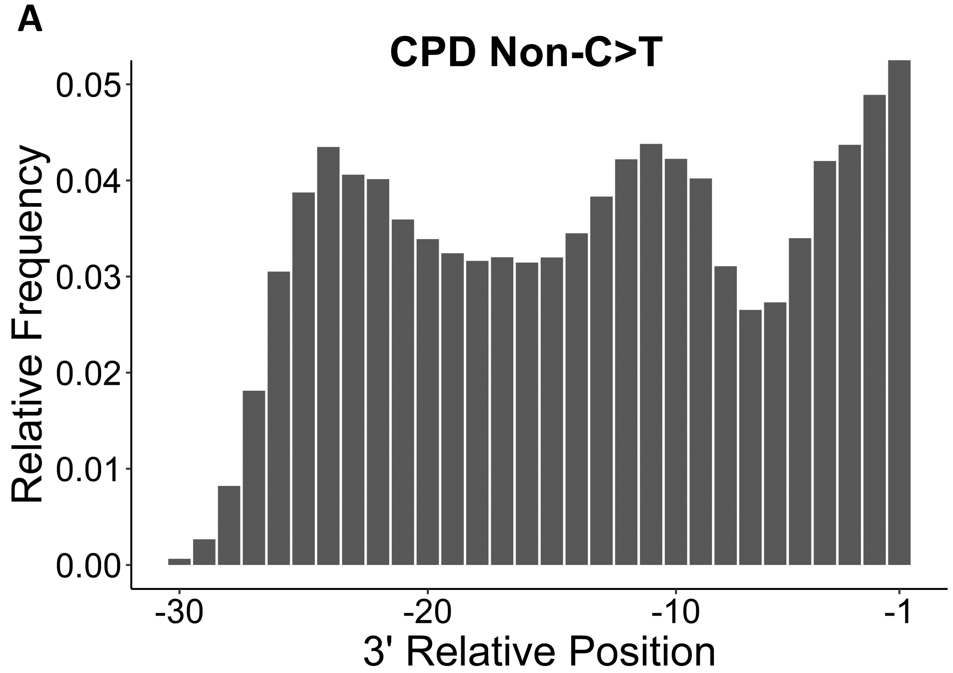

Figure S2: Non-C>T mismatch positions are not enriched ~5-8 nucleotides from the 3' fragment end. (**A**) Frequencies of the occurrence of C>T mismatches at read positions relative to the 3' end. Position -1 on the x-axis represents the first nucleotide before the 3' cut site. Data were derived from CPD XR-seq reads from NHF1 cells after 1h repair.

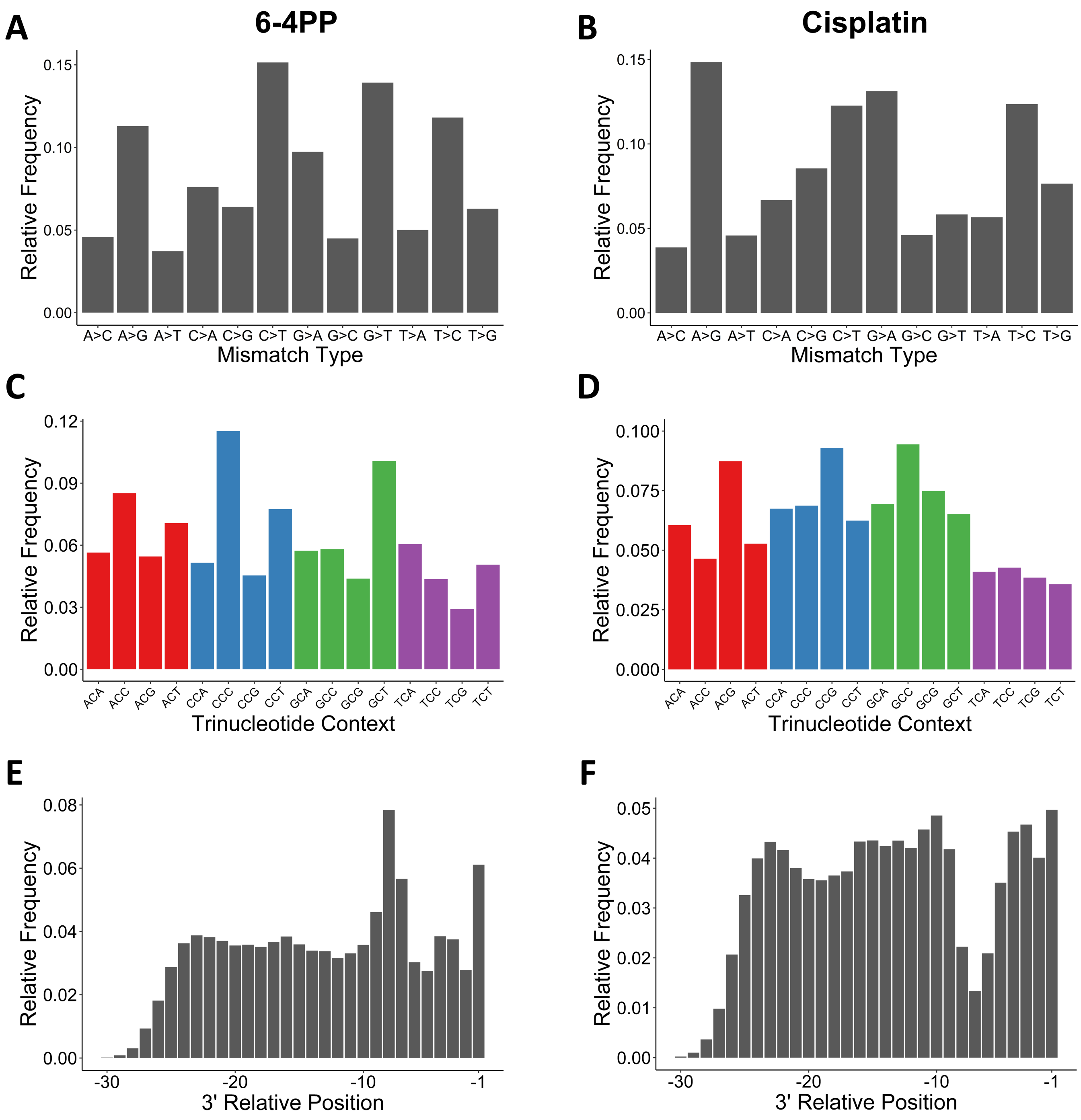

Figure S3: Mismatch data for XR-seq reads derived from repair of 6-4PP and cisplatin adducts are inconsistent with patterns of CPD formation. (**A,B**) Frequencies of each of the 12 single nucleotide mismatch types. (**C,D**) Frequencies of each of the 16 trinucleotide contexts for C>T mismatches. (**E,F**) Frequencies of the occurrence of C>T mismatches at read positions relative to the 3' end. Position -1 on the x-axes represents the first nucleotide before the 3' cut site. Data were derived from 6-4PP XR-seq reads from NHF1 cells after 20min repair (A,C,E) and from cisplatin XR-seq reads from GM12878 cells after 90min repair (B,D,F).

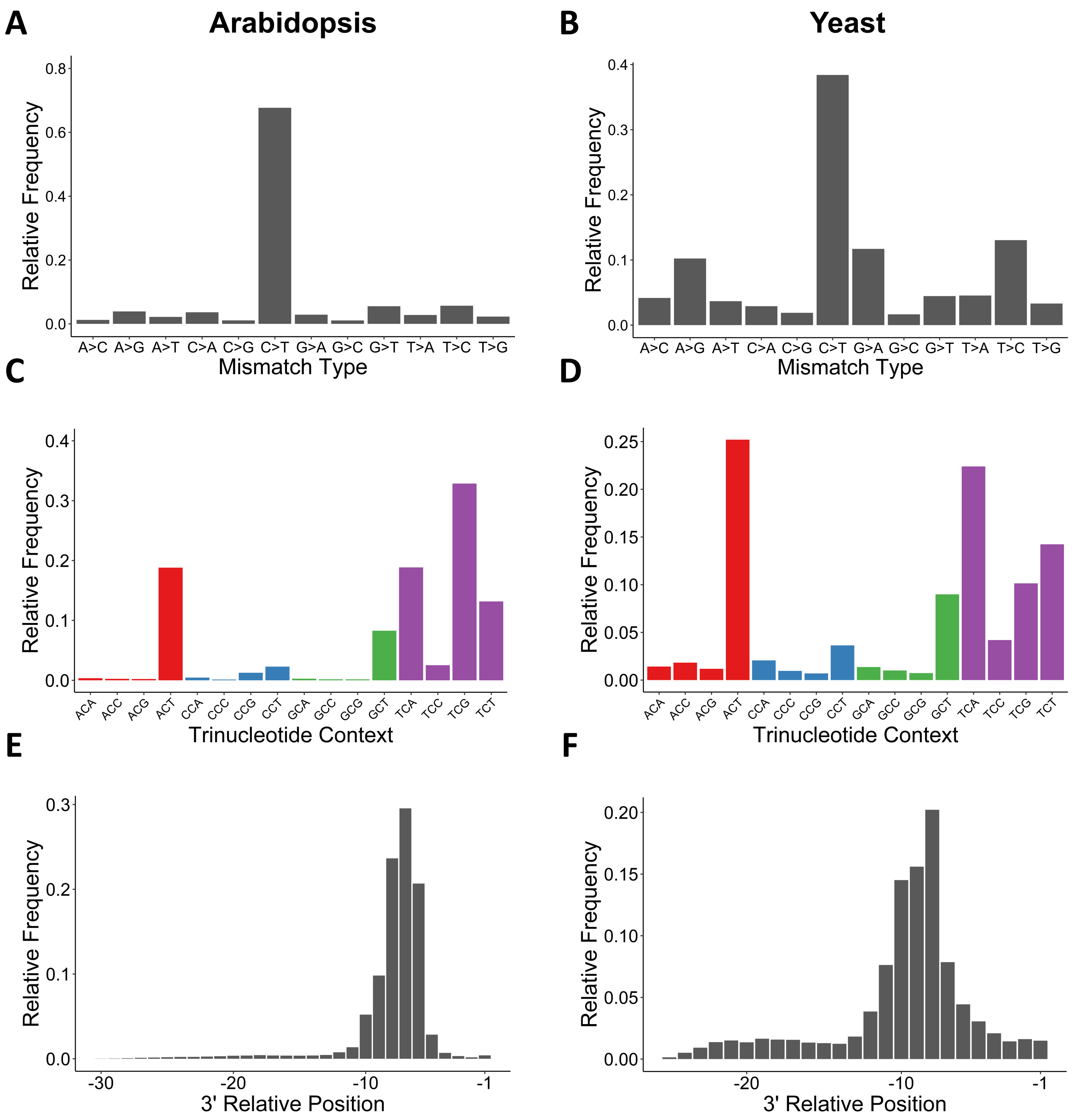

Figure S4: C>T mismatches in aligned XR-seq reads are indicative of CPD positions across eukaryotic species. (**A,B**) Frequencies of each of the 12 single nucleotide mismatch types. (**C,D**) Frequencies of each of the 16 trinucleotide contexts for C>T mismatches. (**E,F**) Frequencies of the occurrence of C>T mismatches at read positions relative to the 3' end. Position -1 on the x-axes represents the first nucleotide before the 3' cut site. Data were derived from CPD XR-seq reads from *Arabidopsis* (A,C,E), and yeast (B,D,F) cells after 30min and 20min repair, respectively.

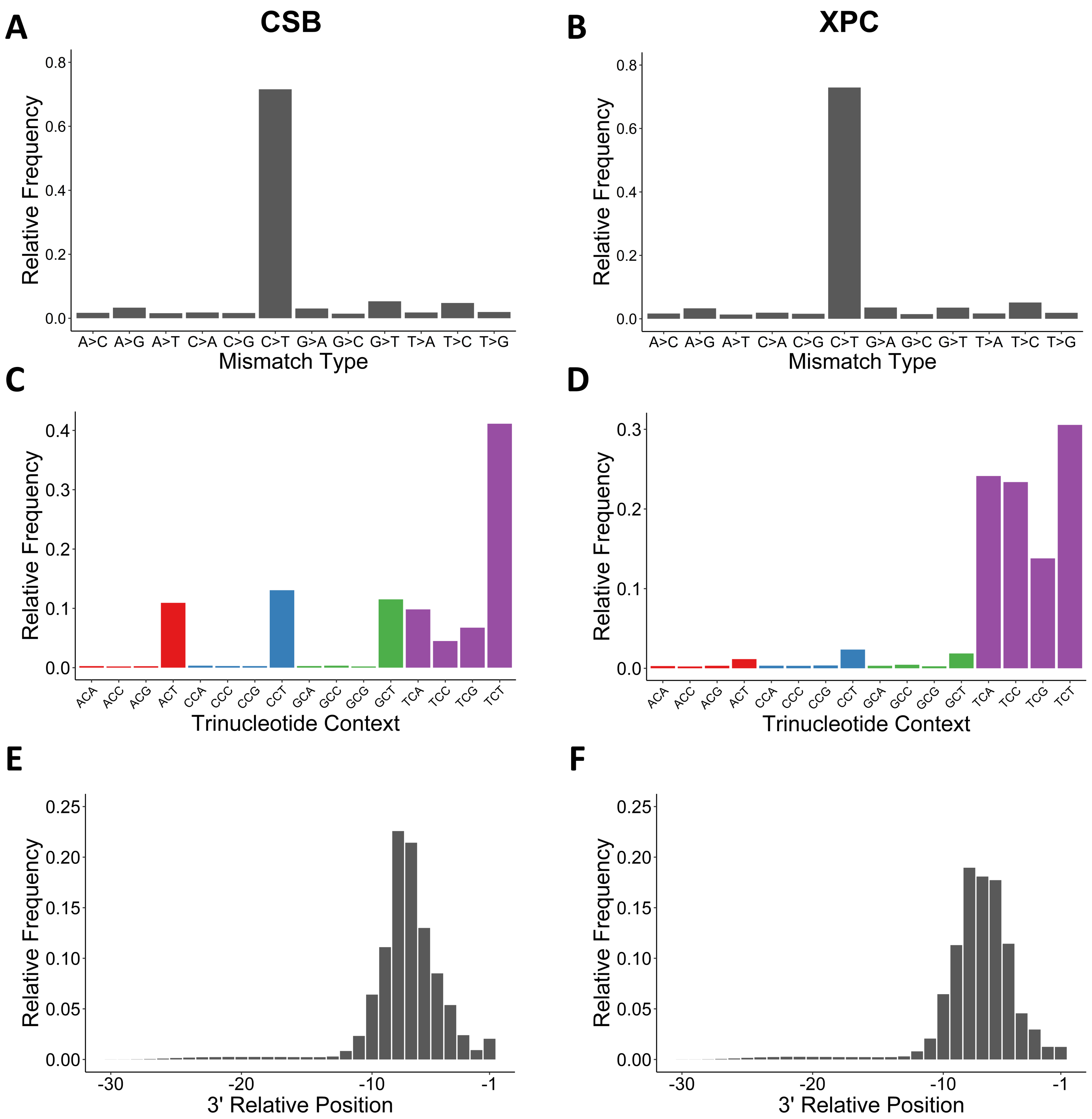

Figure S5: C>T mismatches in aligned XR-seq reads are indicative of CPD positions in either TC-NER or GG-NER defective cells. (**A,B**) Frequencies of each of the 12 single nucleotide mismatch types. (**C,D**) Frequencies of each of the 16 trinucleotide contexts for C>T mismatches. (**E,F**) Frequencies of the occurrence of C>T mismatches at read positions relative to the 3' end. Position -1 on the x-axes represents the first nucleotide before the 3' cut site. Data were derived from CPD XR-seq reads from TC-NER-deficient (CSB; A,C,E) and GG-NER-deficient (XPC; B,D,F) human cells after 1h repair.

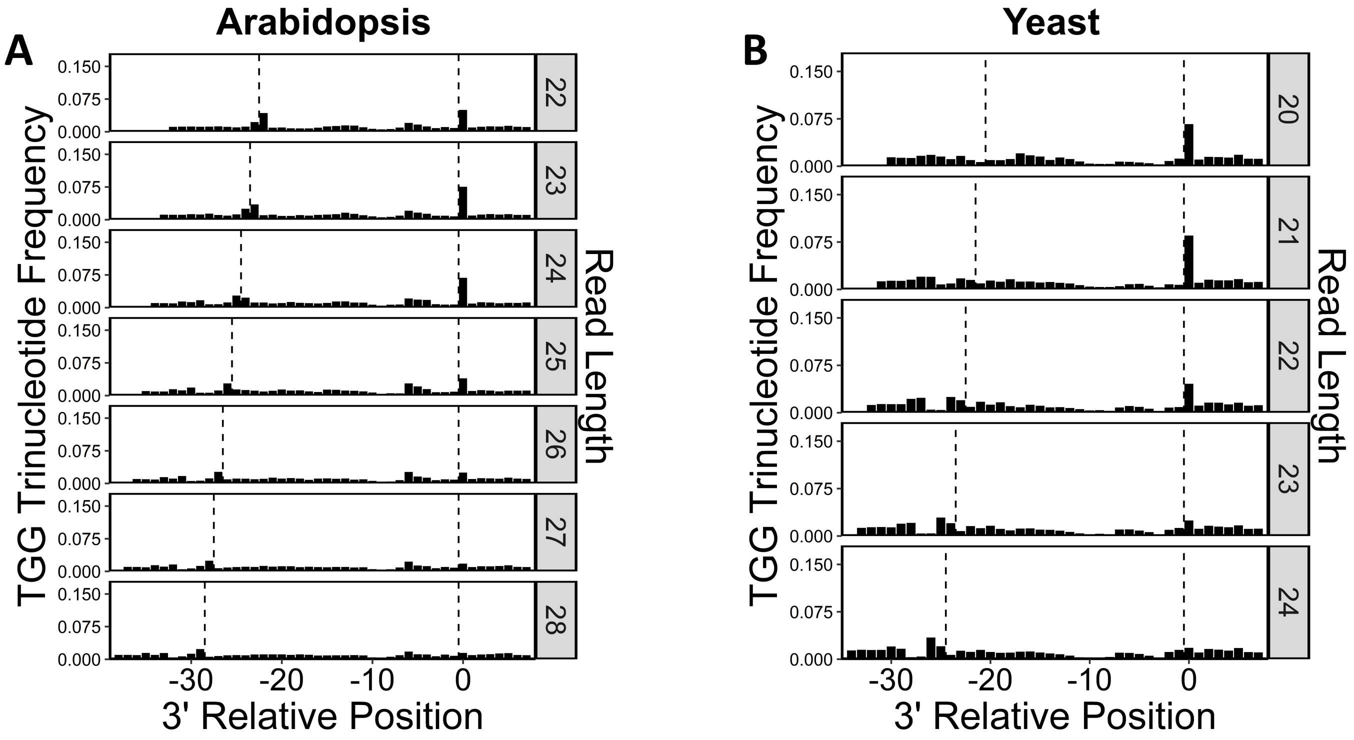

Figure S6: TGG sequences are enriched in Genomic sequences immediately after the 3’ cut site of shorter aligned XR-seq reads derived from *Arabidopsis* and Yeast. (**A,B**) Frequency of TGG sequences in all reads, regardless of mismatches, stratified by read length. Dashed lines represent the 5’ and 3’ NER cut sites, as determined by the ends of reads after adapter trimming. Positions are relative to the thymine in the TGG sequence. Position -1 on the x-axes represents the first nucleotide before the 3’ cut site. Data were derived from CPD XR-seq reads from *Arabidopsis* (A), and yeast (B) cells after 30min and 20min repair, respectively.

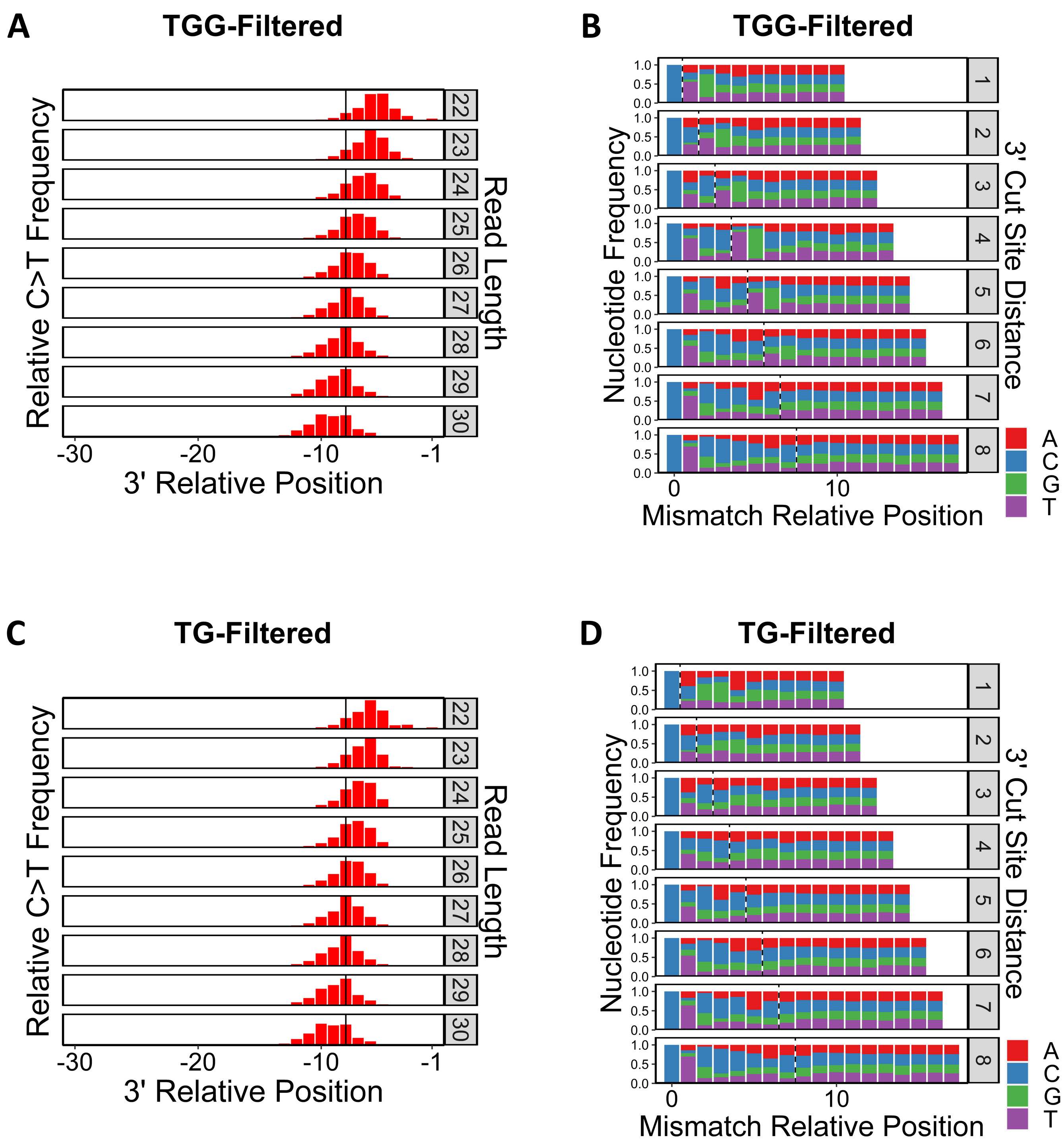

Figure S7: Filtering on adapter prefixes reduces flanking sequence bias and 3' proximal mismatch positions. (**A,C**) Relative frequencies of the occurrence of C>T mismatches at read positions relative to the 3' end, stratified by read length. Bars in red represent significantly upregulated positions (greater than four standard deviations above the mean of a background sample of the ten positions closest to the 5' end; p<6.3e-5). The solid line indicates the most frequent mismatch position across all reads. Position -1 on the x-axes represents the first nucleotide before the 3' NER cut site. (**B,D**) Nucleotide frequencies relative to a 5'-anchored C>T mismatch, stratified by the distance from the mismatch to the perceived 3' NER cut site. The dashed line represents the 3' NER cut site, as determined by the 3' read end after adapter trimming. Position 0 on the x-axes represents the C>T mismatch. All data were derived from CPD XR-seq reads from NHF1 cells after 1h repair and were filtered to remove reads with a 3' flanking TGG (A,B) or TG (C,D) sequence.

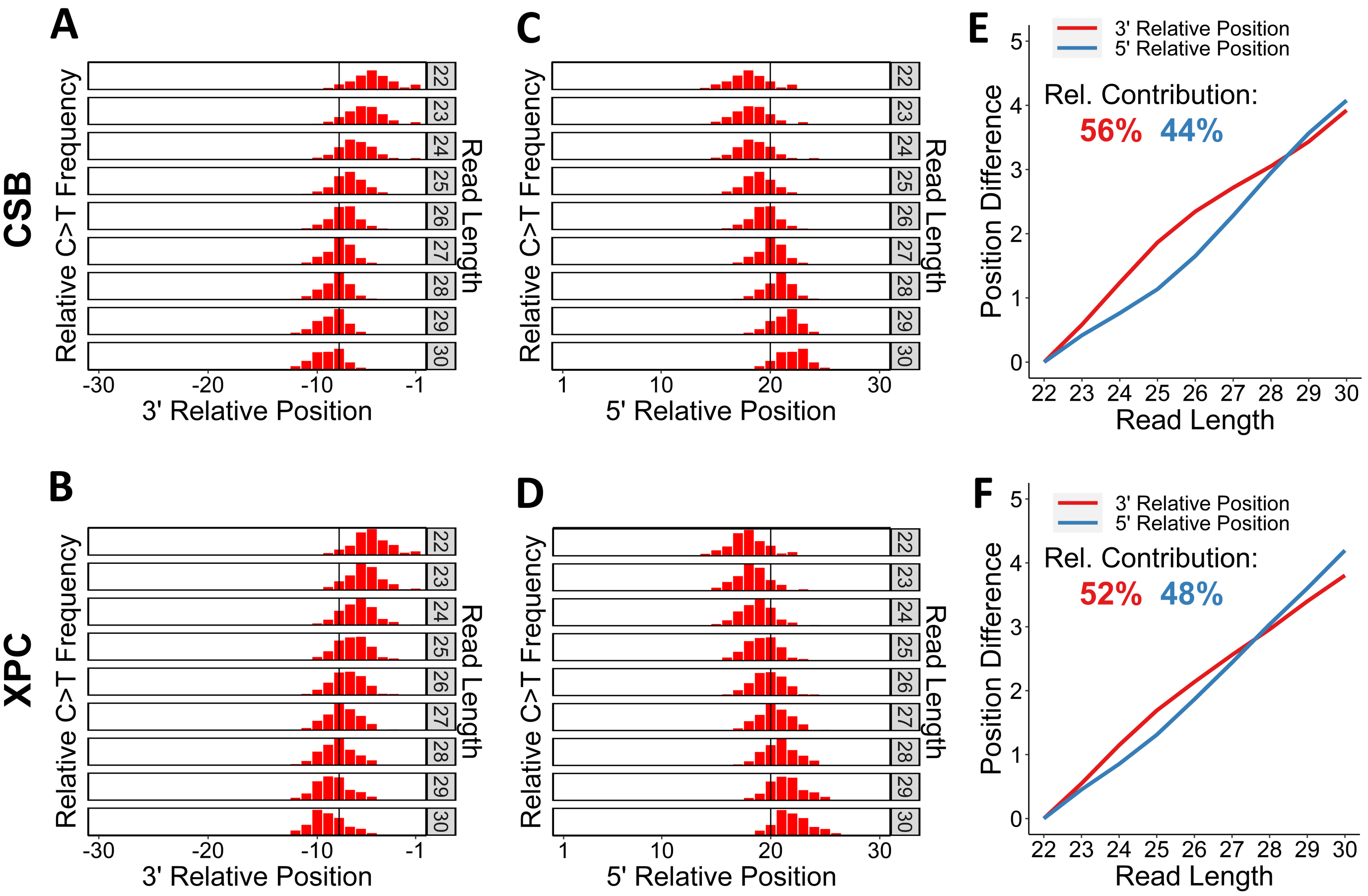

Figure S8: Human NER fragment extension patterns are consistent across different repair backgrounds. (**A-D**) Relative frequencies of the occurrence of C>T mismatches at read positions relative to the 3’ (A,B) and 5’ (C,D) cut sites, stratified by read length. Bars in red represent significantly upregulated positions (greater than four standard deviations above the mean of a background sample of the ten positions closest to the 5’ end; p<6.3e-5). The solid line indicates the most frequent mismatch position across all reads. Positions -1 or 1 on the x-axes indicate the first nucleotide before (A,B) or after (C,D) the related NER cut site, respectively. (**E,F**) Relative 5’ and 3’ contributions to read length variability. Position difference is calculated from the mean C>T mismatch position for a given read length and the mean for the shortest read length. Rel. (Relative) contribution is calculated using the weighted sum of the position difference values where weights are determined by the relative frequencies of each read length. Data were derived from CPD XR-seq reads from TC-NER-deficient (CSB; A,C,E) and GG-NER-deficient (XPC; B,D,F) human cells after 1h repair. For all data, reads with a flanking 3’ TGG sequence were filtered out.

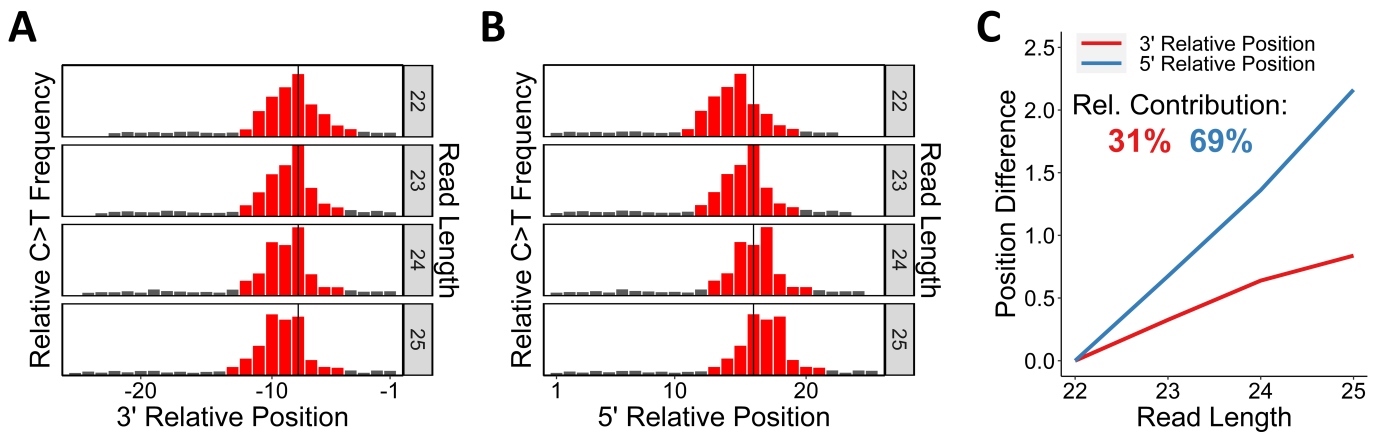

Figure S9: Yeast NER fragment extension is more variable on the 5' end. (**A,B**) Relative frequencies of the occurrence of C>T mismatches at read positions relative to the 3' (A) and 5' (B) cut sites, stratified by read length. Bars in red represent significantly upregulated positions (greater than four standard deviations above the mean of a background sample of the ten positions closest to the 5' end; $p<6.3e-5$). The solid line indicates the most frequent mismatch position across all reads. Positions -1 or 1 on the x-axes indicate the first nucleotide before (A) or after (B) the related NER cut site, respectively. (**C**) Relative 5' and 3' contributions to read length variability. Position difference is calculated from the mean C>T mismatch position for a given read length and the mean for the shortest read length. Rel. (Relative) contribution is calculated using the weighted sum of the position difference values where weights are determined by the relative frequencies of each read length. All data were derived from CPD XR-seq reads from yeast after 20min repair and were filtered to remove reads with a 3' flanking TGG sequence.

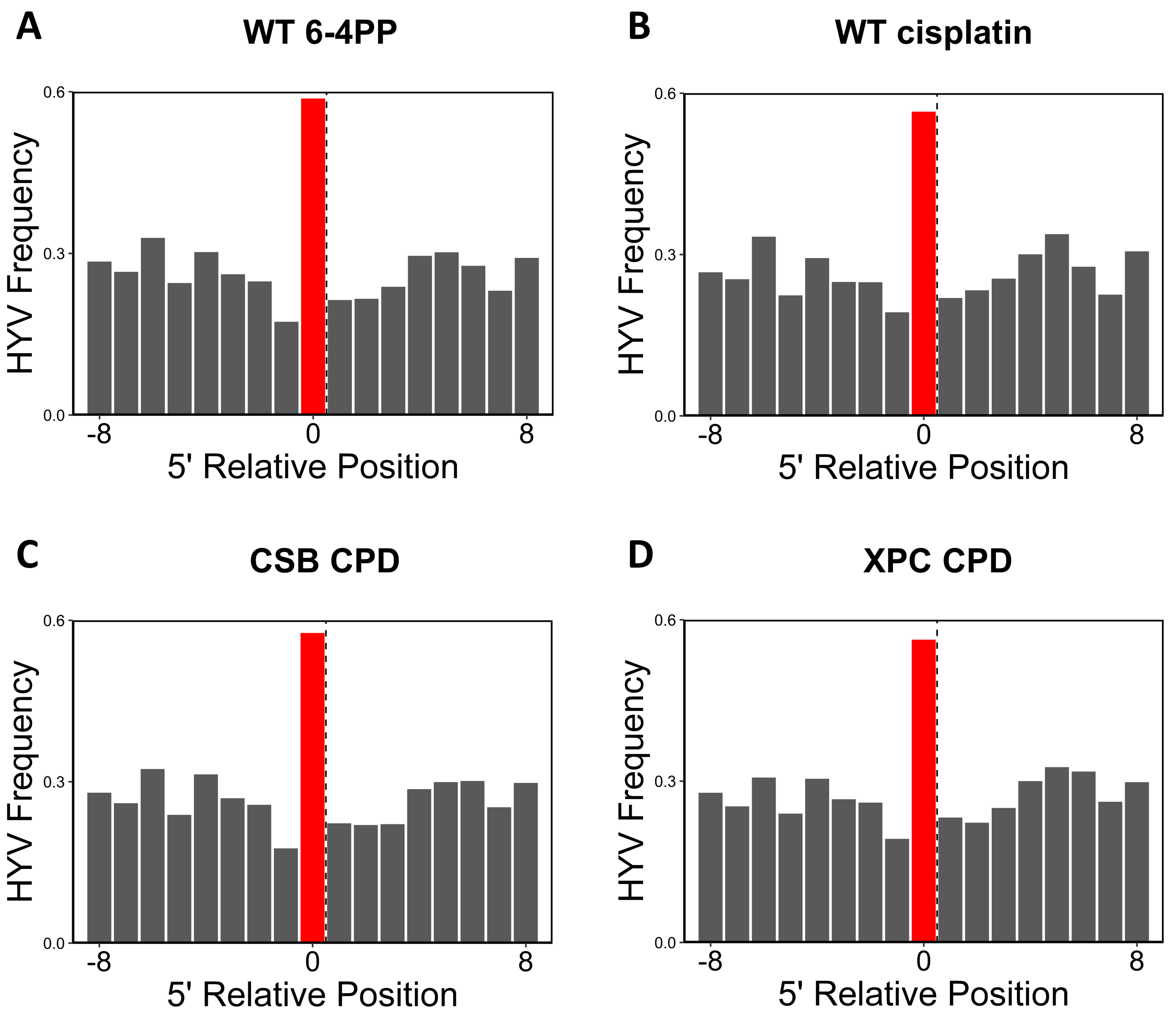

Figure S10: The HYV (not G; C/T; not T) sequence is enriched at the 5' incision site in eukaryotic XR-seq reads, regardless of lesion type or repair background. (**A-D**) Relative HYV sequence frequency in human XR-seq reads with 6-4PP (A), cisplatin (B), or CPD (C,D) lesions and in WT (A,B), TC-NER-deficient (CSB; C) or GG-NER-deficient (XPC; D) repair backgrounds. Data includes all XR-seq reads for the respective cohort, regardless of mismatch content. The dashed line represents the 5' NER cut site. Positions are relative to the pyrimidine in the HYV sequence. Bars in red represent significantly upregulated positions (greater than four standard deviations above the mean of a background sample of the six 5'-most positions; $p<6.3e-5$). Position 1 on the x-axes represents the first nucleotide after the 5' cut site. Data were derived from 6-4PP XR-seq reads from NHF1 cells after 20min repair (A), cisplatin XR-seq reads from GM12878 cells after 90min repair (B), and CSB and XPC mutant cells after 1h repair (C,D). For all data, reads with a flanking 3' TGG sequence were filtered out.

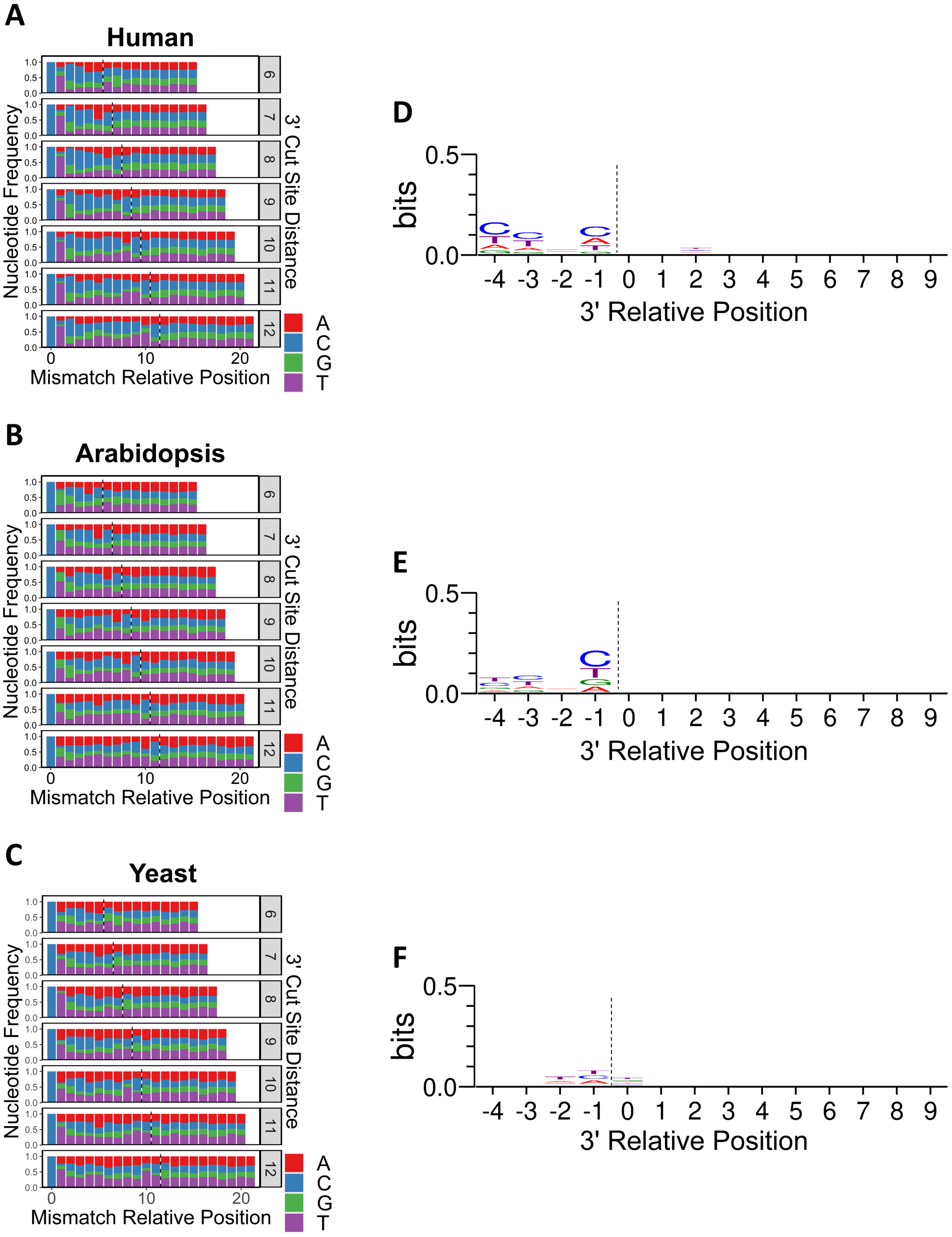

Figure S11: There is no conserved sequence pattern at the 3' incision site across different eukaryotes. (**A-C**) Nucleotide frequencies relative to a 5'-anchored C>T mismatch, stratified by the distance between that mismatch and the 3' NER cut site. Position 0 on the x-axes represents the C>T mismatch. (**D-F**) sequence logos with positions relative to the 3' NER cut site. The information content is normalized to the GC content of input read sequences. Position -1 on the x-axes represents the first nucleotide before the 3' NER cut site. Note that the y-axis is shortened from the maximum information content of 2 bits in order to emphasize the relevant patterns. Dotted lines represent 3' NER cut sites. Data were derived from CPD XR-seq reads from NHF1 (human) cells after 1h repair (A,D), *Arabidopsis* after 30min repair (B,E), and yeast after 20min repair (C,F). For all data, reads with a flanking 3' TGG sequence were filtered out.

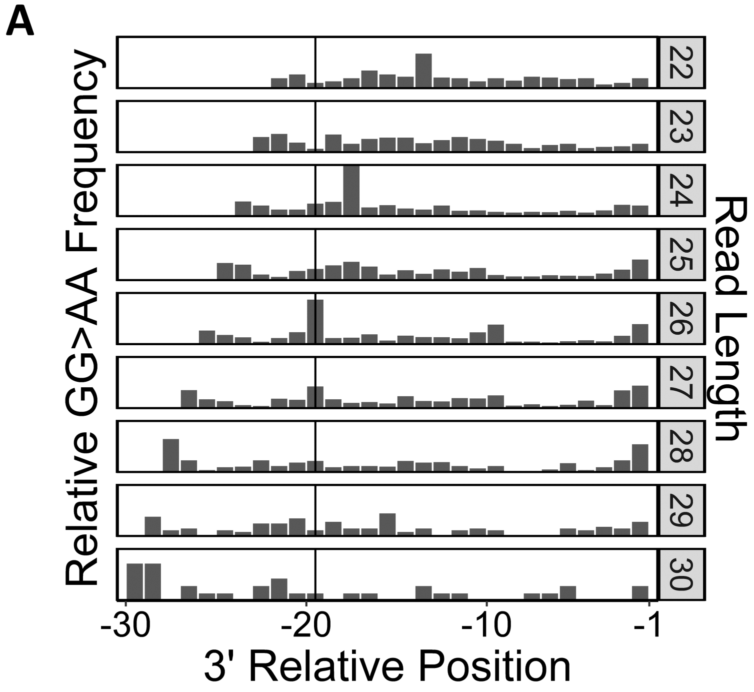

Figure S12: Control GG>AA mismatches in aligned XR-seq reads are not found at positions associated with CPD formation. (**A**) Relative frequencies of the occurrence of GG>AA mismatches at read positions relative to the 3' end, stratified by read length. Bars in red represent significantly upregulated positions (greater than four standard deviations above the mean of a background sample of the ten positions closest to the 5' end; p<6.3e-5). The solid line indicates the most frequent mismatch position across all reads. Position -1 on the x-axis represents the first nucleotide before the 3' cut site. Data were derived from CPD XR-seq reads from NHF1 cells after 1h repair.

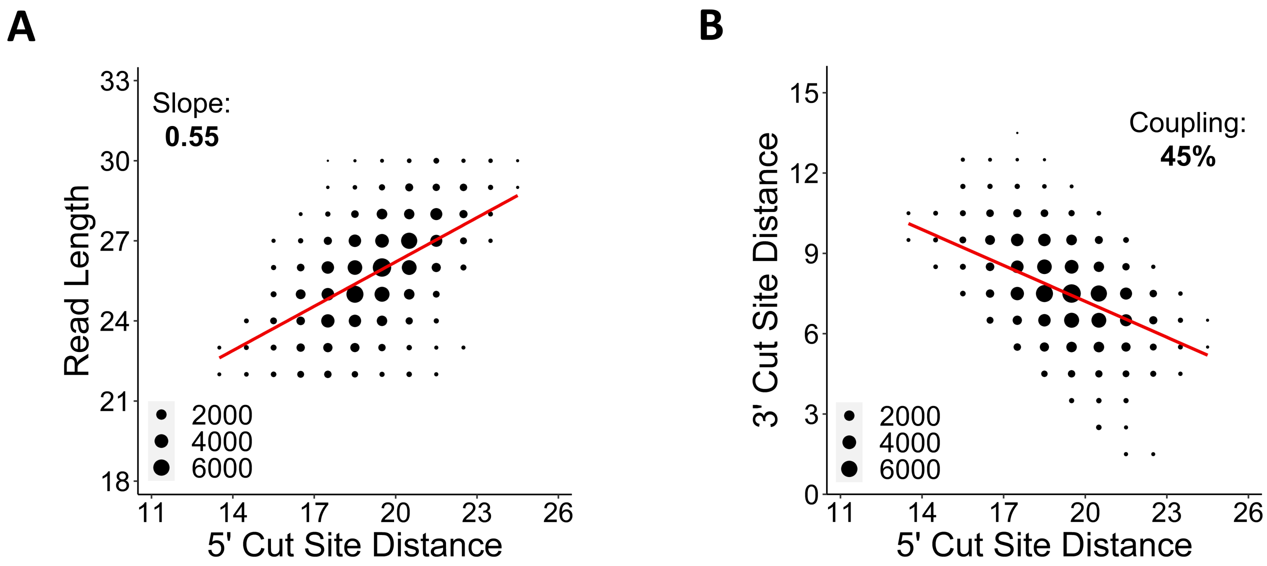

Figure S13: Incision site coupling is consistent across lesion repair timepoints. (**A-B**) Counts of read lengths (A) or 3’ NER cut site distances (B) for each 5’ NER cut site distance. Data were derived from CPD XR-seq reads from NHF1 (human) cells after 8h of repair. Cut site distance was determined using CC>TT mismatch location as a proxy for CPD location. Cut site distance is inclusive of the half-base position between the bases in the CC>TT mismatch. The size of each point on the plot represents the number of XR-seq reads associated with it, as described by the corresponding legend. The red lines represent the linear regression for the data. Coupling percentage was calculated by multiplying the slope of the linear regression by -100% (B). For all data, reads with a flanking 3’ TGG sequence were filtered out.

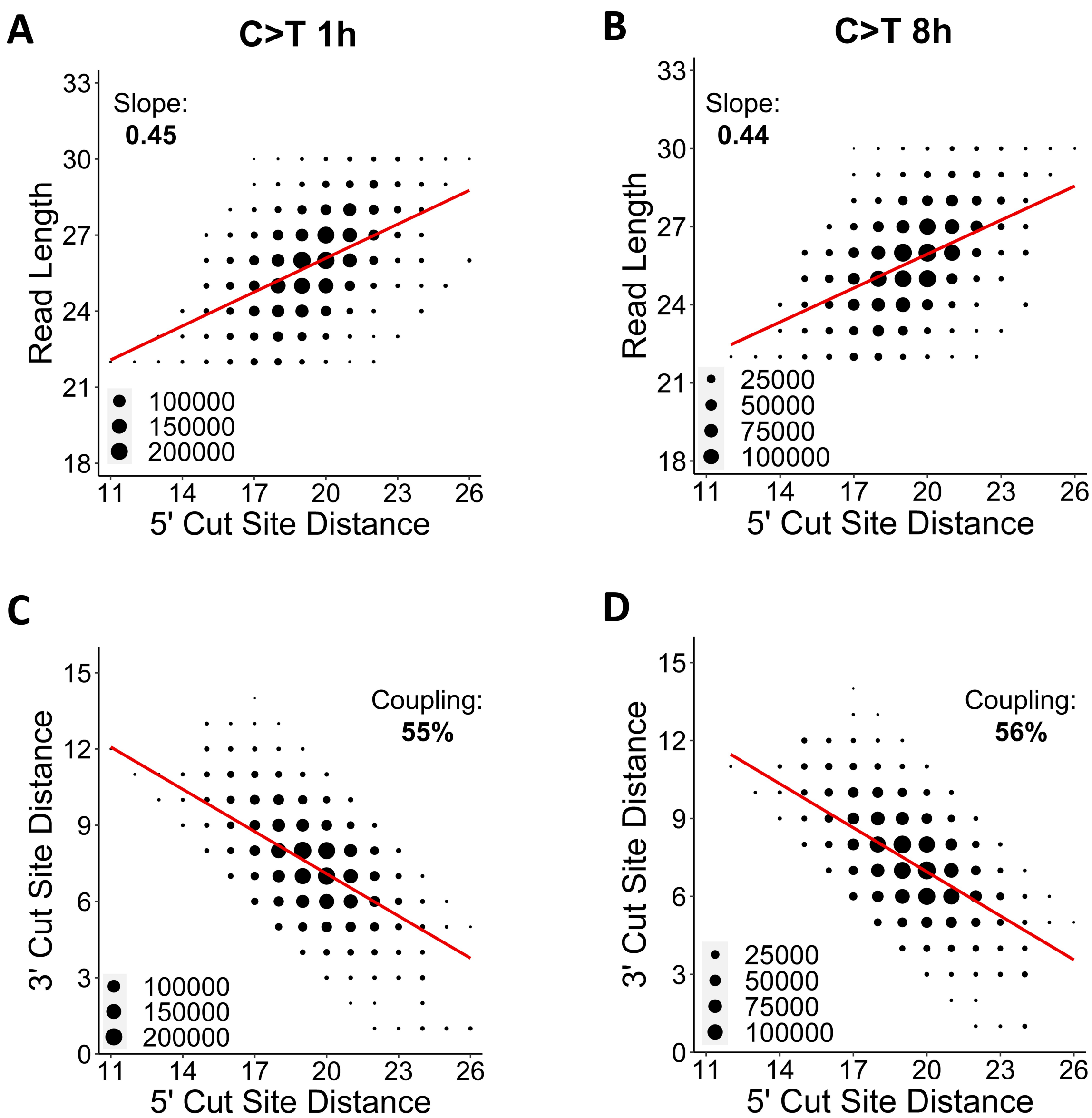

Figure S14: Incision site coupling is slightly higher when single C>T mismatches are used to determine lesion location. (**A-D**) Counts of read lengths (A,B) or 3' NER cut site distances (C,D) for each 5' NER cut site distance. Data were derived from CPD XR-seq reads from NHF1 (human) cells after 1h (A,C) or 8h (B,D) of repair. Cut site distance was determined using C>T mismatch location as a proxy for CPD location. Cut site distance is inclusive of the single in the C>T mismatch. The size of each point on the plot represents the number of XR-seq reads associated with it, as described by the corresponding legend. The red lines represent the linear regression for the data. Coupling percentages were calculated by multiplying the slope of the linear regression by -100% (C,D). For all data, reads with a flanking 3' TGG sequence were filtered out.

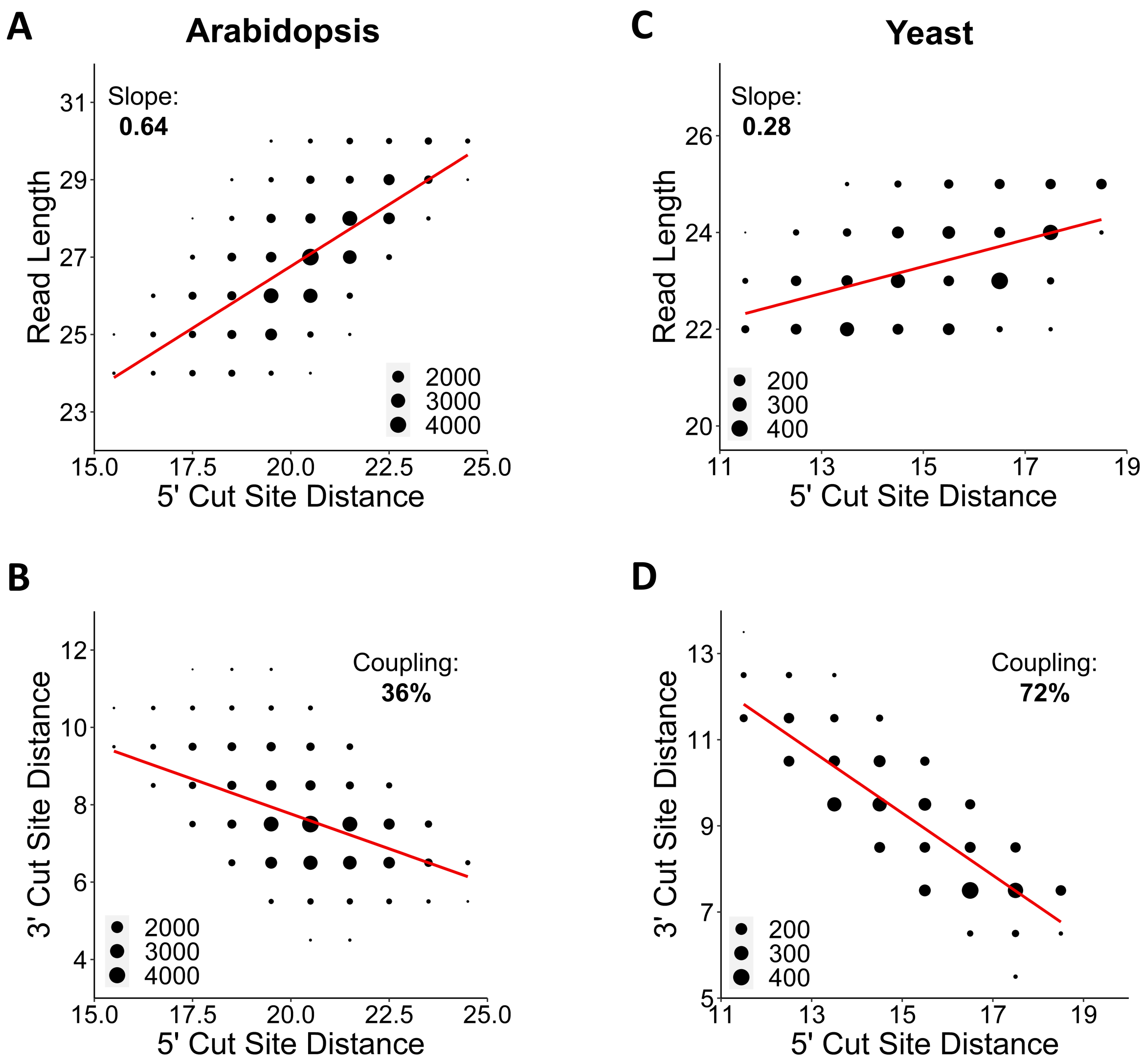

Figure S15: Incision site coupling varies across eukaryotes. (A-D) Counts of read lengths (A,C) or 3' NER cut site distances (B,D) for each 5' NER cut site distance. Data were derived from CPD XR-seq reads from Arabidopsis cells after 30 minutes of repair (A,B) and yeast cells after 20 minutes of repair (C,D). Cut site distance was determined using CC>TT mismatch location as a proxy for CPD location. Cut site distance is inclusive of the half-base position between the bases in the CC>TT mismatch. The size of each point on the plot represents the number of XR-seq reads associated with it, as described by the corresponding legend. The red lines represent the linear regression for the data. Coupling percentages were calculated by multiplying the slope of the linear regression by -100% (B,D). For all data, reads with a flanking 3' TGG sequence were filtered out.

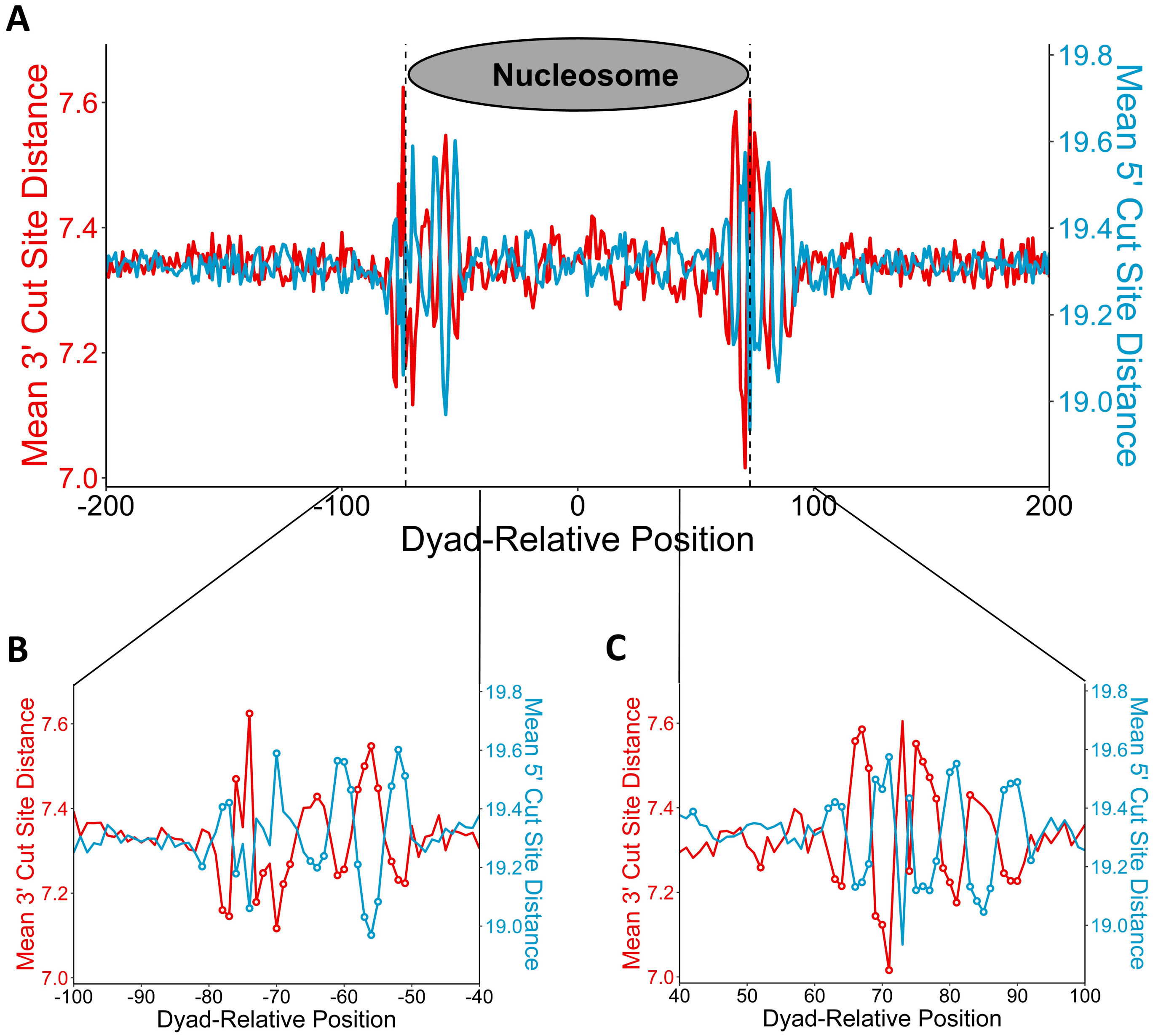

Figure S16: Patterns in incision site selection around nucleosomes are consistent at a later timepoint. (**A**) Mean NER cut site distances for lesions at positions relative to nucleosome dyads. (**B,C**) Highlighted regions from plot A. Cut site distance was derived from single-C>T mismatches treated as CPD lesions and is inclusive of the mismatch position. Dashed lines represent the nucleosome boundaries at positions -73 and 73 (A). Open circles represent positions where the respective cut site distance is significantly above or below the mean ($p < 5e-5$; B,C). Data is strand-aligned so that all 5' incisions occur in the negative direction and all 3' incisions occur in the positive direction, relative to the lesion position. Data were derived from CPD XR-seq reads from NHF1 (human) cells after 8h repair. For all data, reads with a flanking 3' TGG sequence were filtered out.

A

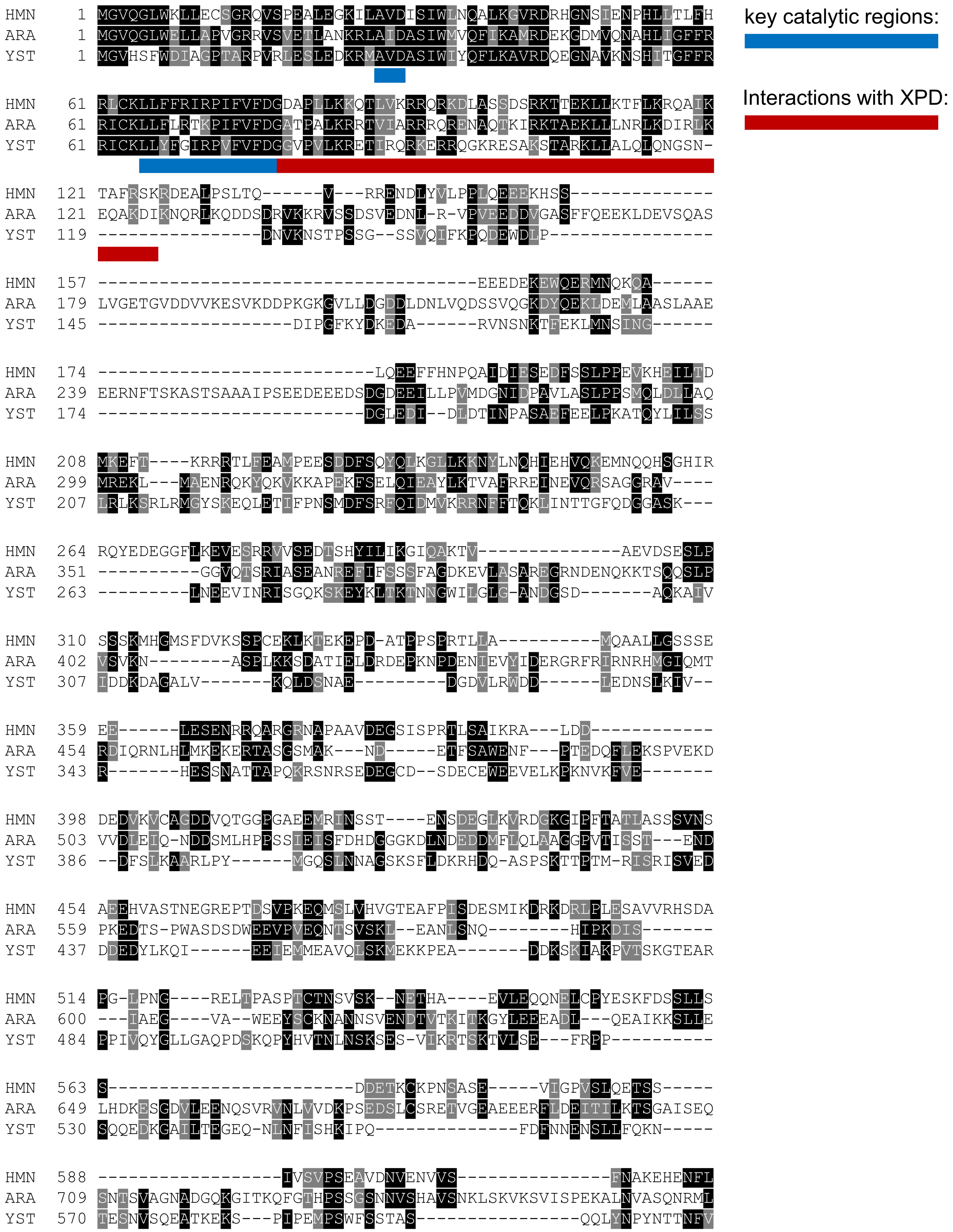
key catalytic regions:
Interactions with XPD:
HMN 1 MGVQGLWKLLECSGRQVSPEALEGKILAVDISIWLNQALKGVRDRHGNSIENPHLLTLFH
ARA 1 MGVQGLWELLAPVGRRVSVETLANKRLAIDASIWMVQFIKAMRDEKGDMVQNAHLIGFFR
YST 1 MGVHSFWDIAGPTARPVRLESLEDKRMAVDASIWIYQFLKAVRDQEGNAVKNSHITGFFR
HMN 61 RLCKLLFFRIRPIFVFDGDAPLLKKQTLVKRRQRKDLASSDSRKTTEKLLKTFLKRQAIK
ARA 61 RICKLLFLRTKPIFVFDGATPALKRRTVIARRRQRENAQTKIRKTAEKLLLNRLKDIRLK
YST 61 RICKLLYFGIRPVFVFDGGVPVLKRETIRQRKERRQGKRESAKSTARKLLALQLQNGSN-
HMN 121 TAFRSKRDEALPSLTQ------V---RRENDLYVLPPLQEEEKHSS--------------
ARA 121 EQAKDIKNQRLKQDDSDRVKKRVSSDSVEDNL-R-VPVEEDDVGASFFQEEKLDEVSQAS
YST 119 ----------------DNVKNSTPSSG--SSVQIFKPQDEWDLP----------------
HMN 157 -------------------------------------EEEDEKEWQERMNQKQA------
ARA 179 LVGETGVDDVVKESVKDDPKGKGVLLDGDDLDNLVQDSSVQGKDYQEKLDEMLAASLAAE
YST 145 -------------------DIPGFKYDKEDA------RVNSNKTFEKLMNSING------
HMN 174 ---------------------------LQEEFFHNPQAIDIESEDFSSLPPEVKHEILTD
ARA 239 EERNFTSKASTSAAAIPSEEDEEEDSDGDEEILLPVMDGNIDPAVLASLPPSMQLDLLAQ
YST 174 --------------------------DGLEDI--DLDTINPASAEFEELPKATQYLILSS
HMN 208 MKEFT----KRRRTLFEAMPEESDDFSQYQLKGLLKKNYLNQHIEHVQKEMNQQHSGHIR
ARA 299 MREKL---MAENRQKYQKVKKAPEKFSELQIEAYLKTVAFRREINEVQRSAGGRAV----
YST 207 LRLKSRLRMGYSKEQLETIFPNSMDFSRFQIDMVKRRNFFTQKLINTTGFQDGGASK---
HMN 264 RQYEDEGGFLKEVESRRVVSEDTSHYILIKGIQAKTV--------------AEVDSESLP
ARA 351 ----------GGVQTSRIASEANREFIFSSSFAGDKEVLASAREGRNDENQKKTSQQSLP
YST 263 ---------LNEEVINRISGQKSKEYKLTKTNNGWILGLG-ANDGSD-------AQKAIV
HMN 310 SSSKMHGMSFDVKSSPCEKLKTEKEPD-ATPPSPRTLLA----------MQAALLGSSSE
ARA 402 VSVKN--------ASPLKKSDATIELDRDEPKNPDENIEVYIDERGRFRIRNRHMGIQMT
YST 307 IDDKDAGALV-------KQLDSNAE---------DGDVLRWDD------LEDNSLKIV--
HMN 359 EE------LESENRRQARGRNAPAAVDEGSISPRTLSAIKRA---LDD------------
ARA 454 RDIQRNLHLMKEKERTASGSMAK---ND-----ETFSAWENF---PTEDQFLEKSPVEKD
YST 343 R-------HESSNATTAPQKRSNRSEDEGCD--SDECEWEEVELKPKNVKFVE-------
HMN 398 DEDVKVCAGDDVQTGGPGAEEMRINSST----ENSDEGLKVRDGKGIPFTATLASSSVNS
ARA 503 VVDLEIQ-NDDSMLHPPSSIEISFDHDGGGKDLNDEDDMFLQLAAGGPVTISST---END
YST 386 --DFSLKAARLPY------MGQSLNNAGSKSFLDKRHDQ-ASPSKTTPTM-RISRISVED
HMN 454 AEEHVASTNEGREPTDSVPKEQMSLVHVGTEAFPISDESMIKDRKDRLPLESAVVRHSDA
ARA 559 PKEDTS-PWASDSDWEEVPVEQNTSVSKL--EANLSNQ--------HIPKDIS-------
YST 437 DDEDYLKQI------EEIEMMEAVQLSKMEKKPEA-------DDKSKIAKPVTSKGTEAR
HMN 514 PG-LPNG----RELTPASPTCTNSVSK--NETHA----EVLEQQNELCPYESKFDSSLLS
ARA 600 ---IAEG----VA--WEEYSCKNANNSVENDTVTKITKGYLEEEADL---QEAIKKSLLE
YST 484 PPIVQYGLLGAQPDSKQPYHVTNLNSKSES-VIKRTSKTVLSE---FRPP----------
HMN 563 S------------------------DDETKCKPNSASE-----VIGPVSLQETSS-----
ARA 649 LHDKESGDVLEENQSVRVNLVVDKPSEDSLCSRETVGEAEEERFLDEITILKTSGAISEQ
YST 530 SQQEDKGAILTEGEQ-NLNFISHKIPQ--------------FDFNNENSLLFQKN-----
HMN 588 ------------------IVSVPSEAVDNVENVVS---------------FNAKEHENFL
ARA 709 SNTSVAGNADGQKGITKQFGTHPSSGSNNVSHAVSNKLSKVKSVISPEKALNVASQNRML
YST 570 TESNVSQEATKEKS---PIPEMPSWFSSTAS----------------QQLYNPYNTTNFV

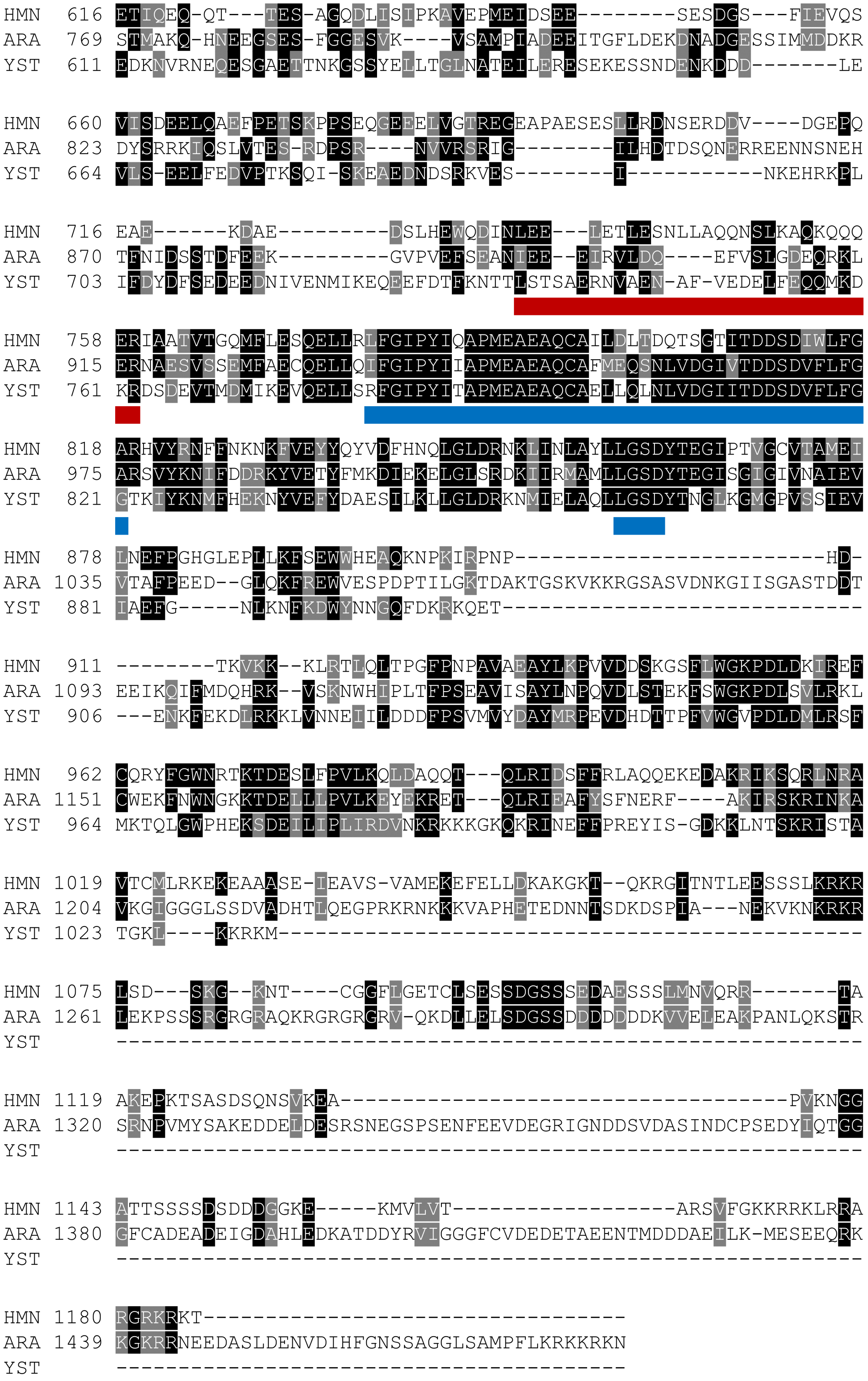

Figure S17: XPG residues which interact with XPD are loosely conserved across eukaryotes. (**A**) Alignment of the amino acid sequences for the 3' NER endonucleases in humans (HMN), Arabidopsis (ARA), and yeast (YST) using the Clustal Omega algorithm. Identically conserved residues are highlighted in black. Partially conserved residues are highlighted in gray. The numbers at the beginning of each line represent the position of the first residue in that line relative to the entire peptide sequence. Blue and Red bars are placed underneath key catalytic regions and XPG helices

which are suspected to interface with XPD, respectively. Amino acid sequences were obtained from the UniProt database (Human: P28715, Arabidopsis: Q9ATY5, Yeast: P07276)
